# Detecting deterrence from patrol data

**DOI:** 10.1111/cobi.13222

**Published:** 2018-11-28

**Authors:** Andrew D. M. Dobson, E. J. Milner‐Gulland, Colin M. Beale, Harriet Ibbett, Aidan Keane

**Affiliations:** ^1^ School of Geosciences University of Edinburgh Edinburgh EH9 3FF U.K.; ^2^ Department of Zoology University of Oxford Oxford OX1 3PS U.K.; ^3^ Department of Biology University of York York Y010 5DD U.K.

**Keywords:** bushmeat, conservation, law enforcement, poaching, protected areas, wild meat, aplicación de la ley, áreas protegidas, carne de caza, carne silvestre, caza furtiva, conservación, 保护, 法律实施, 保护地, 偷猎, 丛林肉, 野味

## Abstract

The threat posed to protected areas by the illegal killing of wildlife is countered principally by ranger patrols that aim to detect and deter potential offenders. Deterring poaching is a fundamental conservation objective, but its achievement is difficult to identify, especially when the prime source of information comes in the form of the patrols’ own records, which inevitably contain biases. The most common metric of deterrence is a plot of illegal activities detected per unit of patrol effort (CPUE) against patrol effort (CPUE‐E). We devised a simple, mechanistic model of law breaking and law enforcement in which we simulated deterrence alongside exogenous changes in the frequency of offences under different temporal patterns of enforcement effort. The CPUE‐E plots were not reliable indicators of deterrence. However, plots of change in CPUE over change in effort (ΔCPUE‐ΔE) reliably identified deterrence, regardless of the temporal distribution of effort or any exogenous change in illegal activity levels as long as the time lag between patrol effort and subsequent behavioral change among offenders was approximately known. The ΔCPUE‐ΔE plots offered a robust, simple metric for monitoring patrol effectiveness; were no more conceptually complicated than the basic CPUE‐E plots; and required no specialist knowledge or software to produce. Our findings demonstrate the need to account for temporal autocorrelation in patrol data and to consider appropriate (and poaching‐activity‐specific) intervals for aggregation. They also reveal important gaps in understanding of deterrence in this context, especially the mechanisms by which it occurs. In practical applications, we recommend the use of ΔCPUE‐ΔE plots in preference to other basic metrics and advise that deterrence should be suspected only if there is a clear negative slope. Distinct types of illegal activity should not be grouped together for analysis, especially if the signs of their occurrence have different persistence times in the environment.

## Abstract 2

*Article impact statement*: Deterrent effects of conservation law enforcement may be robustly assessed by applying relatively simple metrics to ranger patrol data.

## Abstract (es)

Detección de la Disuasión a Partir de Datos de Patrullaje

**Resumen**

La amenaza que representa la caza ilegal de fauna para las áreas protegidas está contrarrestada principalmente por las patrullas de guardias que buscan detectar y disuadir a los delincuentes potenciales. La disuasión de la caza furtiva es un objetivo fundamental de la conservación, pero es difícil identificar cuándo se logra, especialmente cuando la fuente principal de información proviene de los propios registros de las patrullas, que inevitablemente contiene sesgos. La medida más común de la disuasión es una parcela de actividades ilegales detectadas por unidad de esfuerzo de patrullaje (CPUE, en inglés) contra el esfuerzo de patrullaje (CPUE‐E, en inglés). Diseñamos un modelo simple y mecánico del rompimiento y aplicación de la ley en el cual simulamos la disuasión junto con cambios exógenos en la frecuencia de ofensas bajo diferentes patrones temporales del esfuerzo de aplicación. Las parcelas de CPUE‐E no fueron indicadores confiables de la disuasión. Sin embargo, las parcelas de cambio de CPUE sobre cambio en el esfuerzo (ΔCPUE‐ΔE) identificaron con seguridad la disuasión sin importar la distribución temporal del esfuerzo o cualquier cambio exógeno en los niveles de actividad ilegal siempre y cuando el retraso en el tiempo entre el esfuerzo de patrullaje y el cambio en comportamiento subsecuente entre los delincuentes se conocía con cierta aproximación. Las parcelas de ΔCPUE‐ΔE ofrecieron una medida simple y sólida para el monitoreo de la efectividad del patrullaje; no fueron más complicadas conceptualmente que las parcelas básicas de CPUE‐E; y no requirieron de conocimiento de especialistas o algún software para producir. Nuestros hallazgos demuestran la necesidad de dar cuenta de la autocorrelación temporal en los datos de patrullaje y de considerar intervalos apropiados (y específicos a la actividad de caza furtiva) para su agregación. Nuestros hallazgos también revelan vacíos importantes en el entendimiento de la disuasión en este contexto, especialmente para los mecanismos mediante los cuales ocurre. En las aplicaciones prácticas recomendamos el uso de parcelas de ΔCPUE‐ΔE por encima de otras medidas básicas y recomendamos que se sospeche de la disuasión sólo si existe una clara pendiente negativa. No se deben agrupar diferentes tipos de actividades ilegales para su análisis, especialmente si las señales de su ocurrencia tienen diferentes momentos de persistencia en el ambiente.

## 摘要

野生动物的非法捕杀对保护地构成的威胁主要被以发现并阻止潜在罪犯为目标的巡逻队员的巡逻活动来应对。制止偷猎是一项基本的保护目标, 但目标是否实现难以判断, 特别是信息主要来自巡逻队自身记录的时候, 这样就不可避免地存在偏差。判断巡逻威慑力最常见的标准是将每单位巡逻量发现的非法活动 (CPUE) 与巡逻量进行作图 (CPUE‐E) 。我们设计了一个简单机械的违法与执法模型, 模拟了在不同时间格局的执法工作下威慑力与外在因素导致的犯罪频率变化。结果显示, CPUE‐E 图不能可靠地评估巡逻的威慑力。但是对 CPUE 在巡逻量变化下的变化 (ΔCPUE‐ΔE) 作图可以很好地确定威慑力, 且不受巡逻时间分布或非法活动强度外因变化的影响, 只要能大致确定巡逻工作和随后罪犯行为变化的时间差即可。 ΔCPUE‐ΔE 图提供了监测巡逻有效性简单稳健的指标, 它没有比基础的 CPUE‐E 图更难以理解, 也不需要其它专业知识或软件进行绘制。我们的发现指出, 需要考虑巡逻数据的时间自相关性, 并选择适当的 (及偷猎行为特定的) 汇总时间间隔。结果还揭示了目前对威慑力的认识存在不足, 特别是它产生的机制。在实际应用中, 我们建议优先使用 ΔCPUE‐ΔE 图, 且只有斜率明显为负时才应怀疑威慑力的作用。不同类型的非法活动不应该归为一类进行分析, 特别是当它们在环境中留下的痕迹存在时间不一样长的时候。**翻译: 胡怡思; 审校: 聂永刚**

## Introduction

The illegal hunting of wildlife is among the most severe and widespread threats to global biodiversity (Milner‐Gulland et al. 2003; Nasi et al. 2008), and a large proportion of conservation expenditure is directed toward enforcement of wildlife protection laws (e.g., de la Mata & Riega‐Campos 2015; Wright et al. 2016). Ranger patrols are employed in protected areas (PAs) to combat poaching, and an effective patrol strategy is one that leads to fewer instances of illegal activity, whether by incarceration of offenders or by the deterrence of potential future offences (Keane et al. 2011). However, arrest rates of hunters and detection rates of passive hunting techniques, such as snaring, are typically very low in PAs (Watson et al. 2013; O'Kelly et al. 2018a, 2018b), meaning that deterrence is often the dominant means by which ranger patrols are assumed to reduce poaching. Conservationists therefore need to know whether funds spent on ranger patrols actually translate into reductions in the rate of law breaking.

Deterrence is a simple concept but one with complex underlying processes. Early writings on criminal deterrence typically focused on the desire to avoid punishment, but more modern perspectives have widened the definition to include extra‐legal effects (e.g., social censure) as well as strictly economic considerations (such as the loss of criminal opportunity engendered by increased police presence) (Cornish & Clarke 1987; Nagin & Paternoster 1993; Ratcliffe et al. 2011). Regardless of the definition, the existence of deterrence can be difficult to confirm, even with access to long‐term, large‐sample data sets (Paternoster 2010; Nagin 2013). For example, despite the long‐held assumption that police foot‐patrols deter crime, the supporting evidence is weak (Ratcliffe et al. 2011), and numerous studies have found no impact of increasing patrol effort on crime (e.g., Kelling et al. 1974; Bowers & Hirsch 1987; Esbensen 1987). In their randomized controlled trial of foot‐patrol effectiveness in violent crime hotspots in Philadelphia (U.S.A.), Ratcliffe et al. (2011) found a significant reduction in crime target areas after 12 weeks, but the effects were restricted to areas within the top 40% of baseline crime rates.

Critics point out that deterrence is easily confounded with the spatial or temporal displacement of activities that does not cause an overall reduction in their frequency (Reppetto 1976), though there is also evidence of the opposite effect, that of diffusion of crime reduction beyond the policed area, known variously as the halo, free‐rider and free‐bonus effects, among other terms (Weisburd et al. 2006; Braga et al. 2014). Displacement could theoretically also act between crime types, such as burglars switching to drug dealing when homes become more secure, but evidence for all forms of displacement is limited (Weisburd et al. 2006; Guerette & Bowers 2009). Despite such challenges, criminologists have nonetheless been able to identify deterrence in a wide range of situations, including the threat of imprisonment to enforce fine payments (Weisburd et al. 2008) and the implementation of hot‐spot policing to reduce neighborhood crime (Braga et al. 2014).

There has been a lack of consensus on how to infer deterrence when analyzing the effectiveness of ranger patrolling, not least because the context poses specific challenges. For example, wildlife crimes are not independently reported by the victim (as opposed to robbery, theft, or assault). Instead they are discovered only by the patrols themselves, meaning that control areas can rarely be incorporated into studies of deterrence. Independent snare surveys are sometimes undertaken, but these require substantial investment, and there are still challenges with factors such as variable snare detectability (O'Kelly et al. 2018a). Various alternative approaches have been used, including indirect inference from levels of local bushmeat consumption (Hodgkinson 2009), hunter interviews (Gandiwa 2011; St. John et al. 2015), and correlations between observed incidences of poaching and patrol effort (e.g., Leader‐Williams et al. 1990; Steinmetz et al. 2014; Moore et al. 2018). Unfortunately, each approach has limitations. Reported behavior is not likely to be as strong a proxy for deterrence as observed changes in either behavior or the state of the ultimate target for conservation (wildlife populations; De Nicola & Giné 2014). In reality, questions of patrol effectiveness are therefore usually addressed by analyzing the records collected by ranger patrols or other enforcement agents as they go about their duties (e.g., Hilborn et al. 2006; Jachmann 2008; Johnson et al. 2016).

Law enforcement monitoring software packages, such as MIST (Management Information SysTem) and SMART (Spatial Monitoring and Reporting Tool), are widely and increasingly used worldwide to collate, organize, and present these data (e.g., Pimm et al. 2015; Critchlow et al. 2016; Hötte et al. 2016). Outputs are frequently expressed as the number of illegal activities (encounters with poachers or poaching signs), controlling for patrol effort (usually measured as patrol days, or area covered), termed catch per unit effort (CPUE) (Stokes 2010), but the interpretation of these data is not straightforward. Any form of encounter data, even when collected under rigorously designed sampling protocols, is subject to biases, including variable detection rates across seasons and habitats and between observers (Keane et al. 2011; Critchlow et al. 2015). In patrol data, effort is usually deliberately biased toward areas or times where rangers expect to encounter greatest poaching activity (Stokes 2010; Watson et al. 2013).

Widely used basic metrics from patrol data can be particularly misleading (Keane et al. 2011). Catch per unit effort can decline over time in the absence of any deterrence via several different mechanisms. The simplest is a decrease in poaching activity for reasons unrelated to enforcement. Holmern et al. (2007) suggested that the monthly variation in illegal activity which they detected was driven largely by animal migrations, and hence variation in the availability of prey, and Risdianto et al. (2016) reported similar exogenous fluctuations in the frequency of poaching, this time driven by seasonal changes in demand for meat. A similar effect can come about if the frequency of activities is constant but their detectability falls, for example due to a switch in the methods or timing of hunting (Gibson & Marks 1995; Henson et al. 2016).

Recognizing that CPUE over time is a relatively poor measure of deterrence, some authors argue that detecting a negative correlation between CPUE and patrol effort may be a more robust way of identifying deterrence (e.g., Leader‐Williams et al. 1990; Hilborn et al. 2006). However, this metric is vulnerable to the possibility that both variables show similar sorts of linear trends over time for other reasons, leading to spurious correlation. Furthermore, time‐series data display temporal autocorrelation, violating independence assumptions of standard statistical tests. A final challenge is presented by time lags between cause (patrol presence) and effect (hunter behavioral change), which may not be known and which may not align well with the temporal resolution of data collection (e.g., Hötte et al. 2016).

Given the central role of deterrence in PA management effectiveness, there is an urgent need for methods that can identify whether it is present, and techniques for this purpose must be simple to use and applicable to patrol data if they are to be of practical value to PA managers. Analysis of ranger‐collected CPUE data is unlikely to achieve the performance of more targeted studies involving, for example, the experimental manipulation of patrol effort, but patrol data will typically be the only source of information available to managers, meaning that every effort should be made to maximize its utility. Problems caused by temporal trends could be minimized by differencing—computing the change between consecutive observations (Shumway & Stoffer 2017)—potentially yielding a more robust version of the CPUE‐effort plot without the need for complicated analysis. The paucity of independent data against which patrol data may be validated provides an obstacle to investigating options for more this robust metric, which can be overcome using model‐derived simulated data (Zurell et al. 2010). We used a simple model of poacher‐patrol interactions to compare CPUE‐effort plots (hereafter basic plots) with their ΔCPUE‐ΔE counterparts (hereafter differenced plots) in terms of their ability to identify deterrence. We stress tested the metrics by adding exogenous changes in the level of illegal activity at the same time as enforcement‐induced changes, thereby simulating processes (e.g., economic factors) separate from law enforcement that may affect law‐breaking behavior.

## Methods

The appraisal of a potentially biased observation process requires independent, unbiased data. Here we used a highly simplified model of poaching and patrolling in a protected area to simulate time series that comprised poacher‐generated illegal activities, levels of patrol effort, and patrol‐generated CPUE data. We put aside spatial considerations and treated the PA as 1 unit for the sake of simplicity. The model was run in discrete time steps, with and without deterrence. Deterrence was characterized as a reduction in the rate of illegal activity caused by an increase in patrol effort. We also simulated exogenous changes (linear increases or decreases) in the appearance of illegal activities. We generated basic and differenced CPUE‐effort plots for all simulated data and assessed their ability to detect deterrence. Initially, evidence of illegal activities committed in each time step was assumed to disappear at the end of the time step and hence was not detectable in subsequent time steps. We ran further simulations in which detectability persisted to varying degrees and reappraised CPUE‐effort plot performance. In all cases, we assumed an individual activity could only be detected once (in reality there may be multiple detections of a given activity, but patrol records should be able to distinguish between new and previously detected activities).

### Model

The number of illegal activities, *A*, available to be detected by patrols at time *t* is given by Eq. (1), where *A_t_* is the product of the number of hunters and their rate of committing illegal activities in time *t*, added to the number of activities still available to be detected from the previous time step:

(1) $$A_{t}=\alpha_{t}H+\left[p\left({A_{t-}}_{1}-{D_{t-}}_{1}\right)\right],$$

where *H* is the number of potential poachers, *α_t_* is the number of illegal activities carried out per poacher at time *t* (default = 5), *D_t_*_−1_ is the number of detected activities at time *t* − 1, and *p* is the persistence rate of the evidence of activities between time steps (default = 0).

Evidence of illegal activities is detected and removed according to Eq. (2), in which *D_t_* is a saturating function of effort:

(2) $$D_{t}=\left[1-\left({\left(1-z\right)}^{Et}\right)\right]A_{t},$$

where *z* is the probability of detecting an activity given that a patrol occurs in the immediate vicinity (0 < *z *< 1; default = 0.1), and *E_t_* is the patrol effort at time *t* (e.g., proportion of PA covered by patrol per time step).

There is no obvious empirical default value for *α*; the choice here was arbitrary but had no impact on the results. Absolute values of *E* in the model were also arbitrary, but they determined the maximum value of *D_t_*/*A_t_*, the total number of snares detected by all patrols per time step as a fraction of those present in the whole area at *E*_max_ (hereafter *δ*). We set appropriate values of effort by estimating *δ* in a real data set (Hötte et al. 2016) and adjusting *E* in the model to obtain a matching value of *δ* (0.06) (Supporting Information). Among the few studies for which it was possible to calculate *δ*, we found 1 instance where it was much higher (Moore et al. 2018), so we assessed the impact of higher values in the sensitivity analysis.

Changes in the abundance of activities are either caused by deterrence (i.e., a relationship between the rate of appearance of activities [*α_t_*] and patrol effort in the previous time step [*E_t_*_−1_]) or by an unspecified exogenous factor (a consistent change in *α_t_* over time). Here a change in the abundance of activities is equivalent to, and conceptually interchangeable with, a change in the number of poachers. We simulated deterrence by multiplying *α_t_* by 1 − *βE_t_*_−1_, where *β* is a scaling parameter (0 < *β *≤ 1) that controls the maximum extent of deterrence; the default value caused a 20% reduction in poaching activity at the maximum patrol effort level. Exogenous changes in the abundance of illegal activities were simulated firstly by increasing or decreasing *α_t_* in a linear manner over time; *α_t_* is multiplied by 1 + [*γ* ^*^(*t*/*t*_max_)] for the exogenous increase and by 1 − [*γ* ^*^(*t*/*t*_max_)] for the decrease, where the default value of *γ* is 0.5 (Supporting Information); and secondly by using a sine curve with peak‐to‐peak amplitude *γ* and period 80 (the number of time steps; see below) to represent a seasonal pattern of change (Supporting Information). The maximum extent of exogenous change was deliberately made greater than the maximum extent of deterrence (±0.5 vs. 0.2) to provide a suitably stringent test of the CPUE‐effort metrics’ abilities to identify the latter in the presence of the former.

### Simulations

Patrol effort is often distributed unevenly over time (e.g., Hilborn et al. 2006; Risdianto et al. 2016). We simulated the detection of illegal activities over 3 distinct effort profiles, each lasting for 80 time steps (an arbitrary number with no implications for the results): stable (no slope), linearly increasing, and linearly decreasing. All had random noise added, but the mean efforts for each profile were the same (0.2); identical profile series were used for each simulation. Coefficient of variation (CV) in the stable profile was 0.08; for the increasing and decreasing profiles CV = 0.2 (Supporting Information). The profiles introduced deliberate temporal autocorrelation, thereby producing a more stringent test for our metrics. For each of the 3 profiles, we ran 8 simulations, encompassing all possible combinations of deterrence and exogenous change. The CPUE was calculated for each time step, and 2 sets of plots were created. The first is the standard plot of CPUE*_t_* against effort*_t_*_−1_ and the second is the plot of differenced CPUE (CPUE*_t_* − CPUE*_t_*_−1_) against differenced effort (effort*_t_*_−1_ − effort*_t_*_−2_).

The default time lag in response by poachers to changes in ranger effort was equivalent to 1 time step. However, we also explored a more realistic scenario where we assumed imperfect knowledge of these lags and therefore compared ΔCPUE with average patrol effort calculated over a moving window of time steps (*q*). For these simulations, effort*_t_* was recalculated as the mean of effort*_t_*_−_ *_(q_*_−1)_: effort*_t_*. In default simulations, *q* = 5. The resultant plots are hereafter referred to as MA (i.e., moving average) plots to contrast with the default (*t* − 1) plots. We also considered the influences of temporal persistence of individual illegal activities between multiple time steps by repeating simulations for different (nonzero) values of *p*.

In the presence of deterrence, we expected ideal plots to show a negative correlation, indicating that the rate of appearance of illegal activities decreased as patrol effort increased; where deterrence was absent, the slope should be 0, since the frequency of illegal activities was independent of effort. The value of the slope under deterrence can be calculated (Supporting Information), and we present these ideal values alongside the results. The higher the *r* ^2^ value, the more reliable the diagnostic. We appraised the 2 metrics according to these criteria.

## Sensitivity Analyses

We repeated simulations for different values of *β* and *γ*, increasing and decreasing each by 10%. We also repeated simulations with greater amplitude of variation (CV = 0.19, 0.28, and 0.25 for stable, increasing, and decreasing profiles, respectively) to assess its impact on CPUE‐effort relationships. The impact of *q*, the width of the window of moving average, was assessed with differenced plots. The range of variation found in our estimates of *δ* in the few published studies available meant that we ran simulations for the full range to assess its impact.

All modeling and analyses were conducted using R software, version 3.2.3 (R Core Team 2017).

## Results

**Figure 1:**
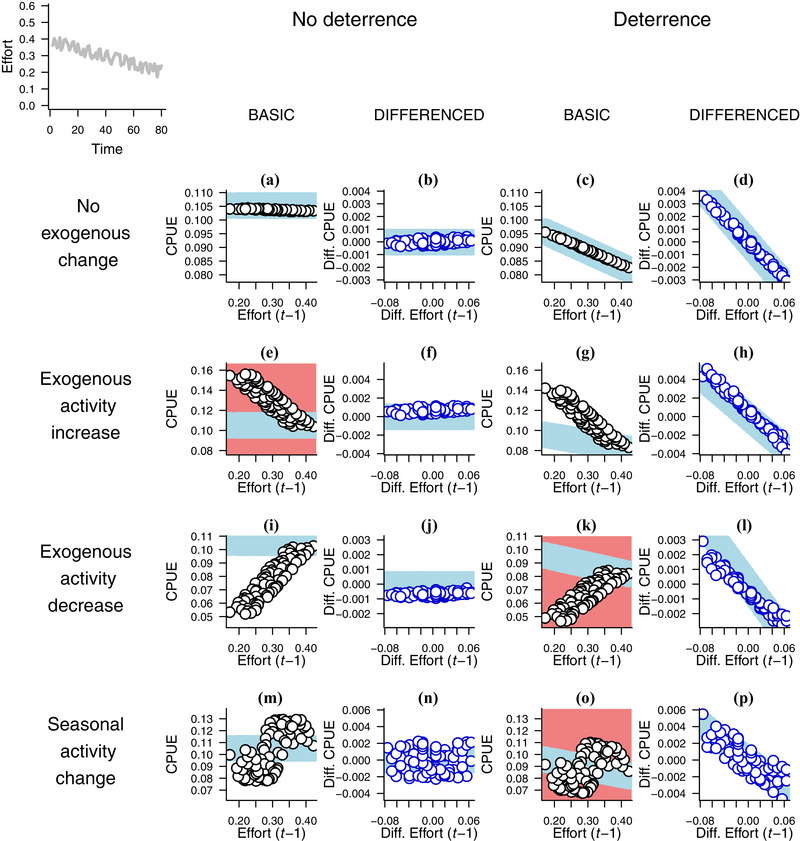
Basic (black circles) and differenced (blue circles) plots (defined in Table 1 footnote) of catch per unit effort (CPUE) against patrol effort in simulated patrol detections of poaching activity under decreasing effort in 8 scenario combinations of deterrence of poaching and exogenous change in poaching activity (*n* = 80 and *t* is 1–80 in each graph). The graph with no letter label in the top‐left shows the effort profile used in the simulations. The blue lines represent the shape of an ideal plot for distinguishing between the presence and absence of deterrence. No‐deterrence slopes are 0, and slopes in deterrence‐graph pairs ([c,d], [g,h], [k,l], and [o,p]) are the same for each member of the pair. The y‐axes are standardized such that deterrence and no‐deterrence pairs have the same range (e.g., [a,c] and [b,d]). Graphs with obvious misattributions have a pale red background; deterrence would incorrectly be inferred in the scenario depicted in (e) and incorrectly missed in (k) and (o). Plots with the stable and increasing effort profiles are in the Supporting Information.

**Figure 2:**
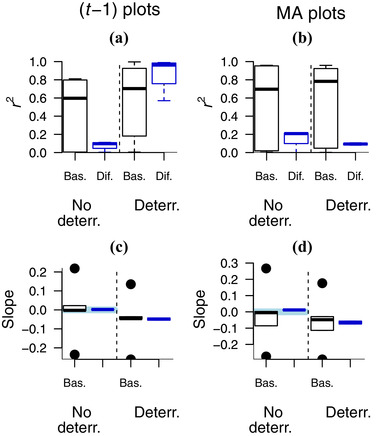
Parameters from basic (black circles and rectangles) and differenced (blue circles and rectangles) plots (defined in Table 1 footnote) of catch per unit effort (CPUE) against patrol effort in simulated patrol detections of poaching activity: (a, c) *t* − 1 plots, poaching activity at time *t* is plotted against patrol effort at *t* − 1; (b, d) MA plots, poaching activity at time *t* is plotted against the average patrol effort in the previous 5 time steps (Bas., basic plots; Dif., differenced plots; no deterr., no deterrence; deterr., deterrence present). In (a) and (b) *r^2^* values are from CPUE‐effort plots across all combinations of patrol effort profile and exogenous change in poaching activity (*n* = 12 for each x‐axis category) with and without deterrence of poaching (whiskers, lowest point within 1.5 interquartile ranges of the lower quartile and highest point within 1.5 interquartile ranges of the upper quartile). In (c) and (d) slope is of CPUE‐effort (green line, ideal values: 0 slope when deterrence is absent [left side of each graph] and negative slope when deterrence is present [right side]). Calculations used to determine the ideal slope are in Supporting Information.

Differenced (*t* − 1) plots consistently returned a clear negative correlation when deterrence was present and a slope close to 0 when it was absent, regardless of the presence or absence of exogenous changes in the appearance of illegal activities, across all effort profiles (Fig. 1 & Table 1). Basic plots yielded at least 1 diagnosis error in each effort profile and yielded far greater variation in both *r* ^2^ values and slope than differenced plots (Fig. 2a and c). Differenced MA plots were less reliable than differenced (*t* − 1) plots for identifying deterrence (compare *r* ^2^, Fig. 2a and b), but the slopes were consistently informative in differenced MA plots and widely variable (hence unreliable) in basic MA plots (Fig. 2d).

**Table 1:** Mean slope and *r* ^2^ values for basic (CPUE‐E) and differenced (ΔCPUE‐ΔE) plots^a^ of illegal activities detected per unit of patrol effort (CPUE) against patrol effort in simulated patrol detections of poaching activity from the 12 scenario combinations of patrol effort profile and exogenous change in poaching

		Deterrence		No deterrence	
Plot type	Effort type^b^	slope (SD)	*r* ^2^ (SD)	slope (SD)	*r* ^2^ (SD)
Basic (CPUE‐E)	*t* − 1	−0.06 (0.14)	0.59 (0.4)	−0.01 (0.17)	0.47 (0.36)
	MA	−0.06 (0.18)	0.56 (0.42)	−0.02 (0.21)	0.55 (0.42)
Differenced (ΔCPUE‐ΔE)	*t* − 1	−0.05 (0.01)	0.89 (0.16)	0 (0)	0.08 (0.05)
	MA	−0.07 (0.02)	0.1 (0.01)	0.01 (0.01)	0.16 (0.1)

a Basic plots are plots of CPUE over patrol effort. Differenced plots are plots of differenced CPUE (CPUE*_t_* − CPUE*_t−_*_1_) over differenced patrol effort (effort*_t−_*_1_ *t* − 1 effort*_t−_*_2_).

b The *t* − 1 plots are those wherein CPUE (or differenced CPUE) at time *t* is plotted against patrol effort (or differenced effort) at time *t* − 1. The MA plots are those wherein CPUE (or differenced CPUE) at time *t* is plotted against the average patrol effort (or differenced effort) across time steps *t* − 4:*t*.

Differenced (*t* − 1) plots had relatively low *r* ^2^‐values when deterrence was absent; median *r* ^2^ was higher for basic plots than differenced plots (Fig. 2a, left of figure & Table 1). This is not apparent from plots of the individual outputs, such as Fig. 1, where graphs (b), (f), and (j) each appear to show an *r^2^* approaching 1 because they were plotted with the same y‐axis limits as graphs (d), (h), and (l) to allow slopes to be compared between deterrence and its absence. This may appear to undermine the superiority of the differenced plots, but an easy rule‐of‐thumb decision can be made: if there is a clear negative slope, deterrence may be operating, and, if not, deterrence is unlikely. No equivalent rule can be formulated for basic plots.

**Figure 3:**
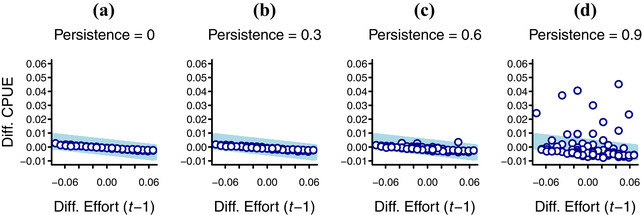
Impact of the persistence of evidence of poaching activity on differenced plots (defined in Table 1 footnote) of catch per unit effort (CPUE) against patrol effort in simulated patrol detections of poaching activity (*n* = 80; *t* is 1–80). The scenario is deterrence of poaching with concurrent exogenous decline in poaching activity under the stable patrol‐effort profile. Graph (a) is equivalent to graph (l) in Fig. 1. The equivalent plots without deterrence and plots with other combinations of exogenous change and effort profile are in Supporting Information.

Increasing persistence in the detectability of illegal activities steadily reduced *r^2^* values in differenced plots (basic plots were not tested) and reduced the steepness of the slope when deterrence was present (Fig. 3 & Table 2), thereby diminishing the ability of the plots to distinguish between the presence and absence of deterrence. This occurred via a simple mechanism. When activities persisted, changes in effort had impacts beyond the consecutive time step, blurring the relationship between effort*_t_* and CPUE*_t+_*_1_ where a relationship was present and adding random noise where it was absent.

**Table 2:** Mean slope and *r* ^2^ values for differenced plots (ΔCPUE‐ΔE)^a^ of illegal activities detected per unit of patrol effort (CPUE) against patrol effort in simulated patrol detections of poaching activity from the 12 scenario combinations of patrol effort profile and exogenous change in poaching under varying persistence times for evidence of poaching

Deterrence		No deterrence		
slope (SD)	*r* ^2^ (SD)	slope (SD)	*r* ^2^ (SD)	Persistence
−0.05(0.01)^b^	0.89 (0.16)	0 (0)	0.08 (0.05)	0
−0.05 (0.01)	0.87 (0.18)	0 (0)	0.01 (0.01)	0.1
−0.05 (0.01)	0.84 (0.2)	0 (0)	0 (0)	0.2
−0.04 (0.01)	0.8 (0.22)	0 (0)	0.03 (0.02)	0.3
−0.04 (0.01)	0.74 (0.24)	0 (0)	0.04 (0.02)	0.4
−0.04 (0.01)	0.65 (0.25)	0 (0)	0.02 (0.02)	0.5
−0.04 (0.01)	0.46 (0.21)	0 (0)	0.01 (0.01)	0.6
−0.04 (0.01)	0.2 (0.12)	0 (0)	0 (0)	0.7
−0.04 (0.01)	0.06 (0.05)	−0.01 (0)	0 (0)	0.8
−0.04 (0.01)	0.02 (0.02)	−0.01 (0)	0 (0)	0.9

a Definitions in Table 1 footnotes.

b The first row is equivalent to row 3 of Table 1.

A related mechanism explains the fact that *r^2^* values of the differenced plots diminished as the width of the window, *q*, for calculating the moving average in MA plots increased from 1 to 4 (Supporting Information); random noise was introduced by the averaging of additional time steps that actually had no predictive value. Sensitivity tests revealed no meaningful influence of changes in *β* or *γ*, or of increased variance in effort profiles, on the relative or absolute performances of basic and differenced *t* − 1 plots, though greater amplitude did cause a slight increase in *r* ^2^ of differenced MA plots, as well as a more negative slope when deterrence was present (Supporting Information). The performance of differenced plots was insensitive to the maximum value of *δ*, the total number of snares detected by all patrols per time step as a fraction of those present in the whole area at *E*_max_ (*D_t_*/*A_t_*); however, interpretation of the differenced plots was easiest for values *δ* ≤ 0.06. As *δ* increased, both the ideal and realised slopes tended toward 0 (i.e., became less negative) under deterrence, making deterrence progressively less clearly identifiable (Supporting Information).

The threshold of 0.06 is a practical rule of thumb as opposed to an essential criterion but, if applied, will effectively impose a constraint on the ratio of time‐step length to patrol effort per time step (for a given detection rate). In the model, the value of *δ* was set with reference to the absolute values of *E*, but when applying the method to real data, the patrol effort will be a known value, meaning that it is the length of the time step that must be deliberately chosen (Supporting Information).

## Discussion

Deterrence is a fundamental aim of conservation law‐enforcement patrols, but it is difficult to identify, such that very few convincing analyses exist that demonstrate deterrence empirically in the field (but see Moore et al. 2018). Observed declines in CPUE over time, which are frequently taken as evidence of deterrence, may be caused by exogenous processes that have little or nothing to do with behavioral responses to patrol effort. Here we demonstrate that basic CPUE‐effort plots, which are often presented as a remedy to this issue, are vulnerable to the same forms of bias, and we show how differenced plots (ΔCPUE‐ΔE) are more informative. Differenced plots reliably distinguished between the presence and absence of deterrence, regardless of the temporal distribution of effort or any exogenous change in illegal activity levels. These plots are no more conceptually complicated than the basic CPUE‐effort plots and require no specialist knowledge or software to produce.

Differenced plots were most effective when the time lag between patrol effort and subsequent behavioral change among offenders was known. If the lag between a patrol and its effect on hunter behavior is equivalent to *x* time steps, then the differenced CPUE should ideally be plotted against the differenced effort *x* time steps earlier. When data were averaged over a longer period to compensate for a potential lack of this knowledge, the plots became much noisier, though they could still be used to distinguish between deterrence and its absence.

Time lags are difficult to specify, given the current state of our understanding of the relationship between enforcement effort and illegal behavior. In the absence of independent data, it might be appropriate under some circumstances to make numerous plots, each at different time lags, and appraise all of them for signals of deterrence, but there is a danger of data‐mining and producing spurious positive results by chance. We recommend choosing a small set of plausible potential time lags to explore, based on poacher interviews, expert judgement or other lines of available evidence.

Persistence also affected the diagnostic ability of the CPUE‐E plots and is likely to exist to some extent in all patrol data. At one extreme, a wire snare may remain in a landscape for many months after the hunter who set it has left the scene (Coad 2007). At the other extreme, a gun‐hunting trip only exists while the hunter is present, and its detectability thereafter depends on the hunter's discarding of empty cartridges or other paraphernalia, some of which may be less obviously associated with a hunt (an exception is the killing of large fauna, where the majority of the carcass is likely to remain). However, persistence may be an easier problem to manage than unknown time lags. The potential solutions are to conduct field experiments to determine how long various signs of illegal activity—from snares and gun cartridges to animal carcasses—remain detectable (fairly simple experiments may suffice) and to aggregate patrol data only across classes of activity that have similar persistence times.

A further complication, not addressed here, is that of spatial displacement. In the context of PA management, 3 broad outcomes of such displacement are possible. First, displacement occurs within the PA (no net reduction in illegal hunting). Second, displacement occurs from the entirety of the PA into surrounding areas of similar, unprotected habitat (hunting pressure may not be reduced). Third, displacement occurs from the entirety of the PA into surroundings that are unsuitable for hunting of the species of concern, but may still be good for other resource uses (displacement effectively constitutes a cessation of conservation‐relevant hunting [i.e., deterrence]). In practice, the second and third outcomes are indistinguishable when no data from outside the PA are available, and the first outcome may not be obvious unless patrols include sufficient variety in their routes (Watson et al. 2013; Critchlow et al. 2015). Investigation of this phenomenon requires a spatially explicit model, ideally with individual agents representing patrols and potential offenders, and programmed responses linking enforcement effort with behavior of the latter. The spatial unit over which data are aggregated must also be considered in light of displacement; where fine‐scale information is lacking, the PA boundary is probably the most parsimonious choice.

There are also factors we did not consider, such as variation in patrol motivation and efficiency, that could either mask or mimic a signal of deterrence, and that merit investigation. The effects of deterrence and other aspects of law enforcement on hunter behavior are also likely to be dependent upon the socioeconomic circumstances of potential law‐breakers, as well as the availability of alternative economic opportunities (Nasi et al. 2008; Cawthorn & Hoffman 2015). In the meantime, wherever patrol data forms the basis of decisions (a common situation, given the rapid trend toward their widespread use for monitoring law‐enforcement effectiveness), we suggest a set of rules of thumb to apply to the analysis and interpretation of patrol data. First, do not use plots of CPUE against effort to determine the presence of deterrence. These plots are liable to yield both false‐positive and false‐negative errors in a wide range of circumstances. Plots of CPUE over time carry even less reliable information. Second, use differenced CPUE‐effort plots. Only suspect deterrence if there is a clear negative slope. Bear in mind that differenced plots work best for low values of *δ*; set the length of the time step over which to aggregate data accordingly. Third, if in doubt, do not interpret changes in CPUE with effort as evidence for deterrence. Be aware of the different persistence times of different types of illegal activity and the interactions between activity persistence in the landscape and the spatial pattern of patrolling. Depending on sample sizes, analysis of distinct types of activity could be made separately, with the caveat that if the analysis is carried out on only 1 type of activity, a simple switch in technology or prey species in response to patrol effort may be mistaken for deterrence. Overlaps in patrols in time and space are also complicating factors which may preclude the use of simple metrics.

**Figure 4:**
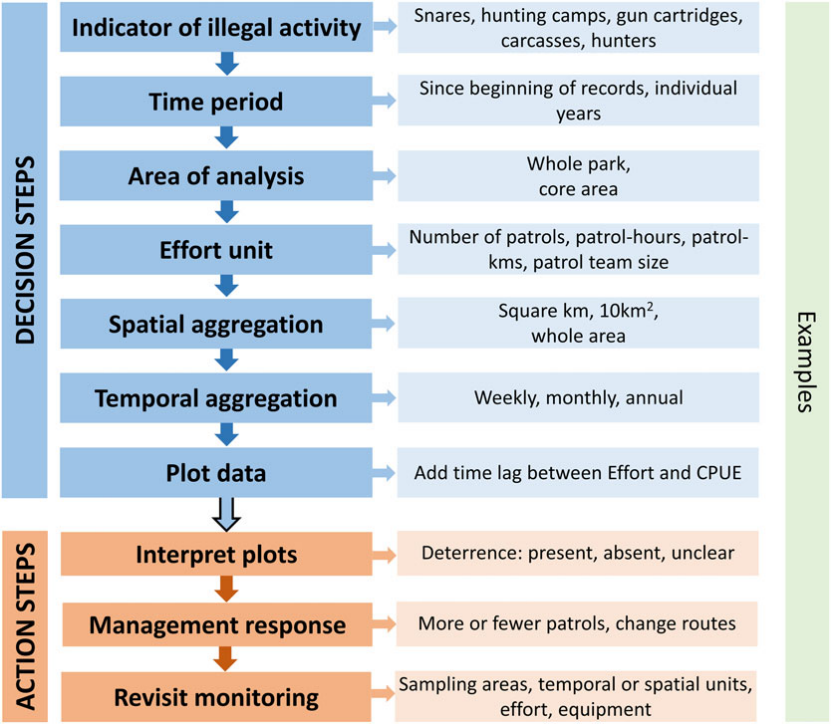
Decisions involved in generating differenced plots (defined in Table 1 footnote) of catch per unit effort (CPUE) over patrol effort from basic antipoacher patrol data and management actions taken as a result.

In practical applications, producing these plots from real patrol data sets will require a number of decisions to be made, including the subset of infractions to be investigated, the time period and area to be investigated, the unit of patrol effort to be used and the level of spatial and temporal aggregation (Fig. 4). Some, such as the choice of time lags, have already been discussed. The choice of effort unit may be guided by hypotheses about the mechanism by which patrol presence causes deterrence. The extents of spatial and temporal aggregation are likely to be influenced by sample size, but the total area and total time frame to be assessed could require careful consideration (see the second rule of thumb, above), especially if effort levels have varied across time and in space. Choices should be based on a substantive understanding of the patrol context and be fully recorded and justified.

A lack of negative slope in the differenced plots does not “necessarily” indicate that there is no deterrent effect of patrolling. Deterrence may occur in a binary fashion: present when there are at least some patrols and absent when there are not. However, the management response to a flat plot might be similar whether or not deterrence of the sort described is suspected; in either case careful consideration should be given as to whether increasing the frequency (or visibility) of patrols is the best way to deter hunters. If deterrence is not realistically achievable, patrol resources could be better directed toward tasks that may not be expected to cause deterrence but that may otherwise be useful, such as clearing snares, or toward different approaches to promoting deterrence, such as intelligence‐led raids.

In the longer run there are no quick fixes to understanding the drivers of success for law enforcement in PAs. The CPUE‐based metrics derived from ranger patrol data cannot replace more detailed studies in which at least some of the potentially confounding factors are either measured or held constant. More, and better, information is needed about the dynamic relationships between ranger and poacher behavior, temporally and spatially, in order to plan and implement effective interventions.

## Supporting information

Supplementary figures (Appendix S1), the calculations used to derive the correct slope in CPUE‐effort plots (Appendix S2), R code for models and plots (Appendix 3), and a spreadsheet of effort values used in the R code (Appendix 4) are available online. The authors are solely responsible for the content and functionality of these materials. Queries (other than absence of the material) should be directed to the corresponding author.

Supporting Information

Supporting Information
