# Supplementary material for: Detecting deterrence from patrol data
Source: Conserv Biol. 2018 Nov 28;33(3):665–75. doi: 10.1111/cobi.13222 (PMC7379181; doi:10.1111/cobi.13222)
Supplement: Supplementary file 1 — Supplementary figures (Appendix S1), the calculations used to derive the correct slope in CPUE‐effort plots (Appendix S2), R code for models and plots (Appendix 3), and a spreadsheet of effort values used in the R code (Appendix 4) are available online. The authors are solely responsible for the content and functionality of these materials. Queries (other than absence of the material) should be directed to the corresponding author. [file COBI-33-665-s001.docx]

# Supporting Information

## Appendix S1. Supplementary figures


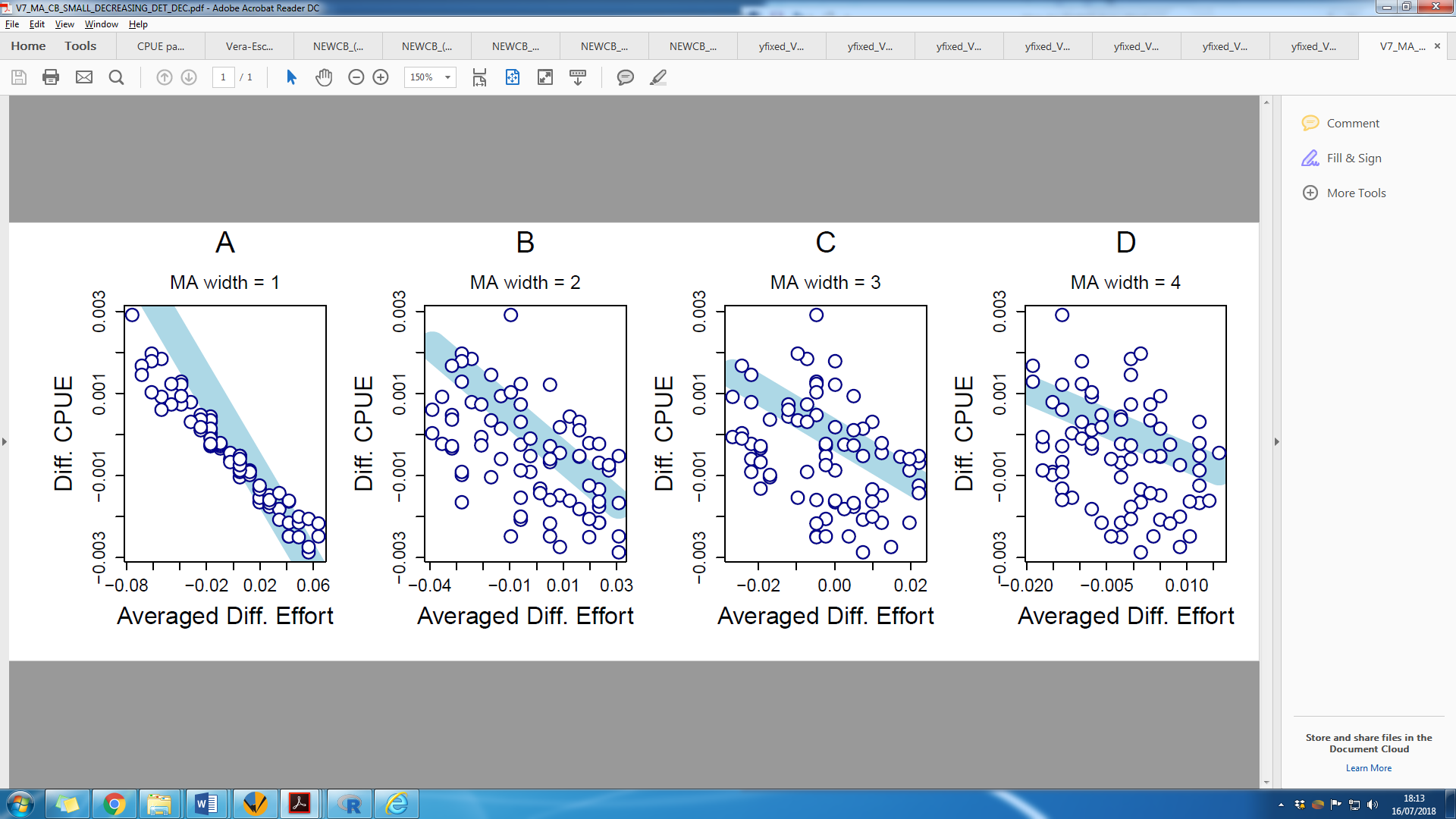


**Fig. S1.** Impact of the width of the moving average ‘window’ on differenced MA CPUE-effort plots (*n*=80). The scenario is deterrence with exogenous decline, under the decreasing effort profile. Note that an MA width of 1 (panel *A*) is equivalent to the (*t*-1) setting; it is therefore the same as Fig. 1*L*.


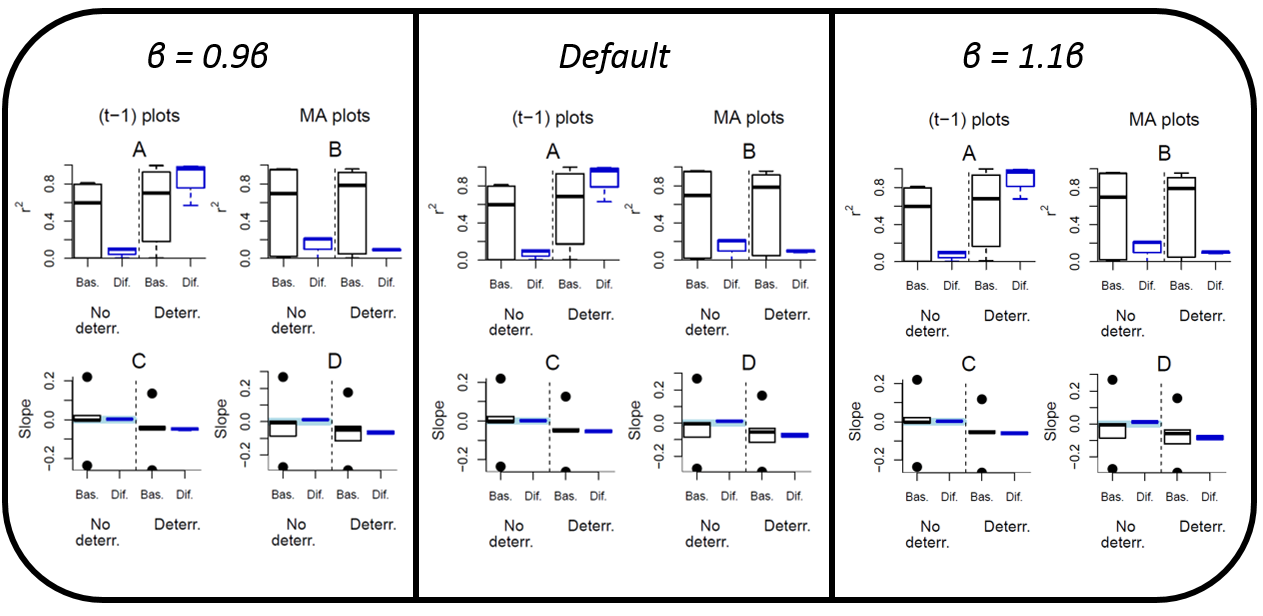


**Fig. S2.** Impact of *β* on slope and *r^2^* of (*t*-1) and MA plots (*n*=9 per panel). The central section is identical to Fig. 2.


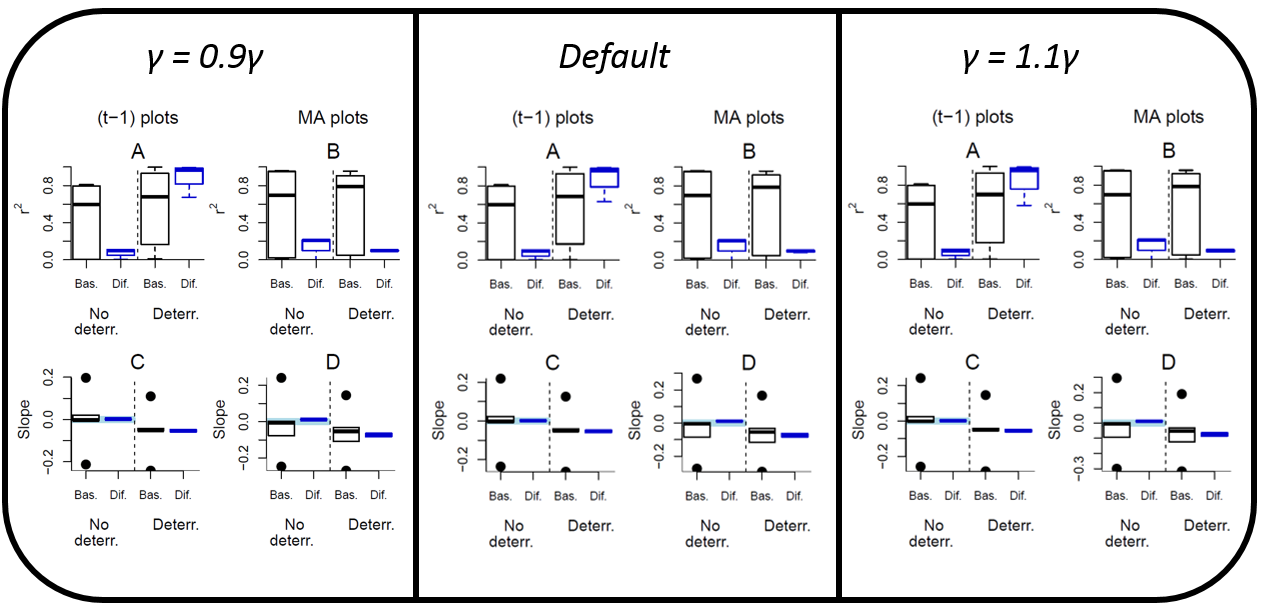


**Fig. S3.** Impact of *γ* on slope and *r^2^* of (*t*-1) and MA plots (*n*=9 per panel). The central section is identical to Fig. 2.


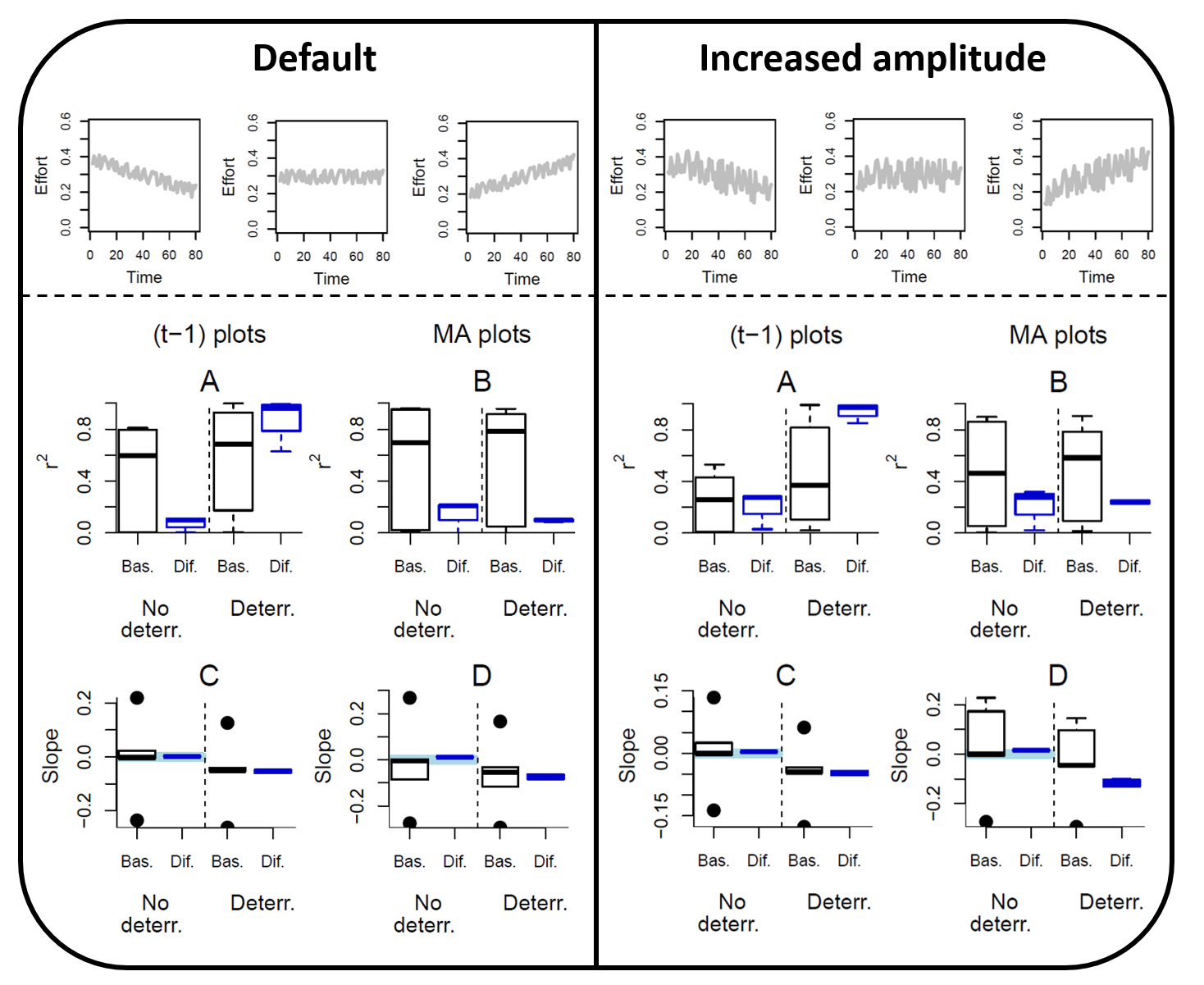


**Fig. S4.** Impact of amplitude of effort profiles on slope and *r^2^* of (*t*-1) and MA plots. The upper sections illustrate the effort profiles used in the respective simulations. The lower left section is identical to Fig. 2.


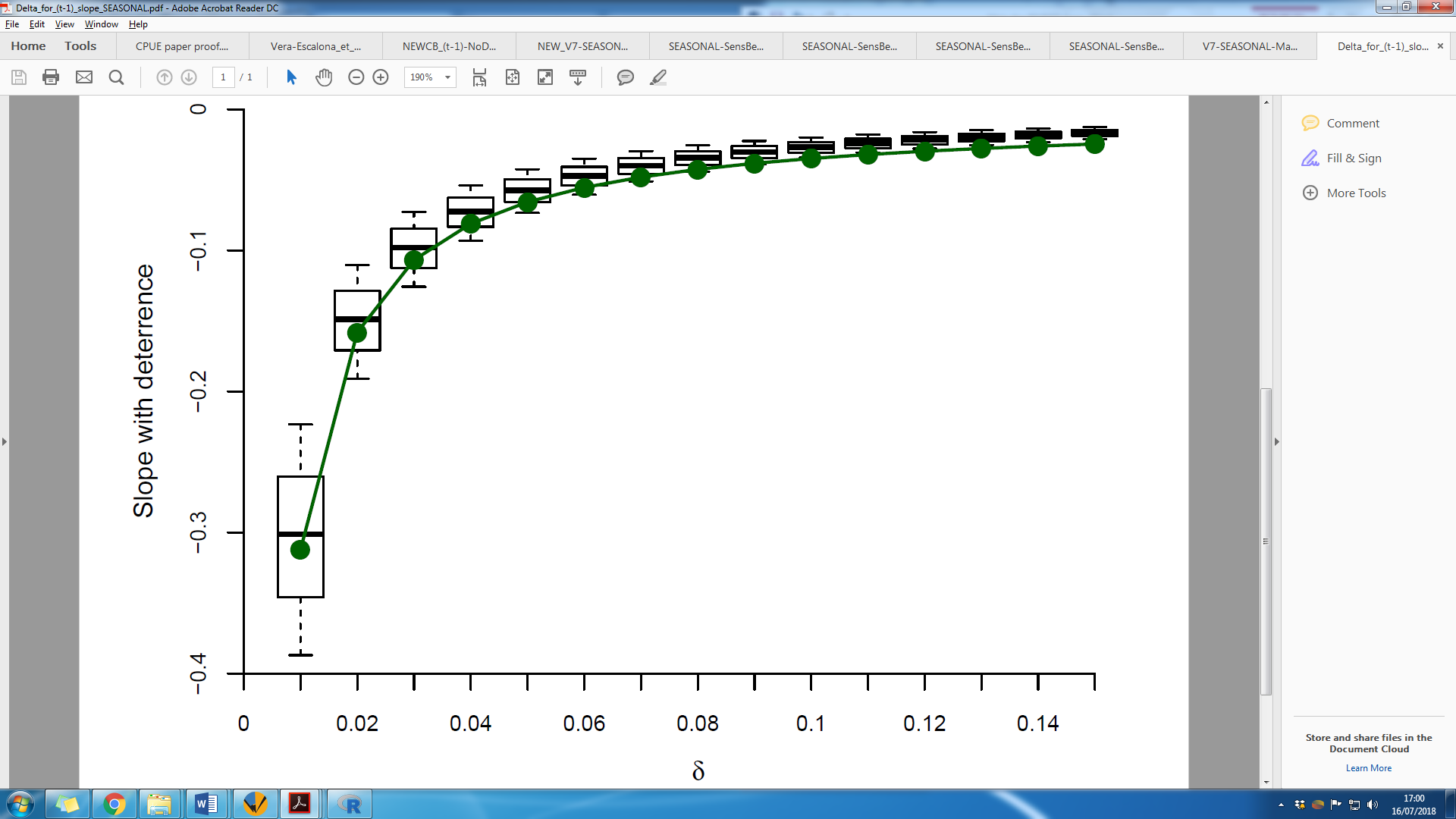


**Fig. S5.** Impact of δ on the slope of differenced (*t*-1) plots under deterrence. Boxplots show model medians; the green points show the ideal slope.


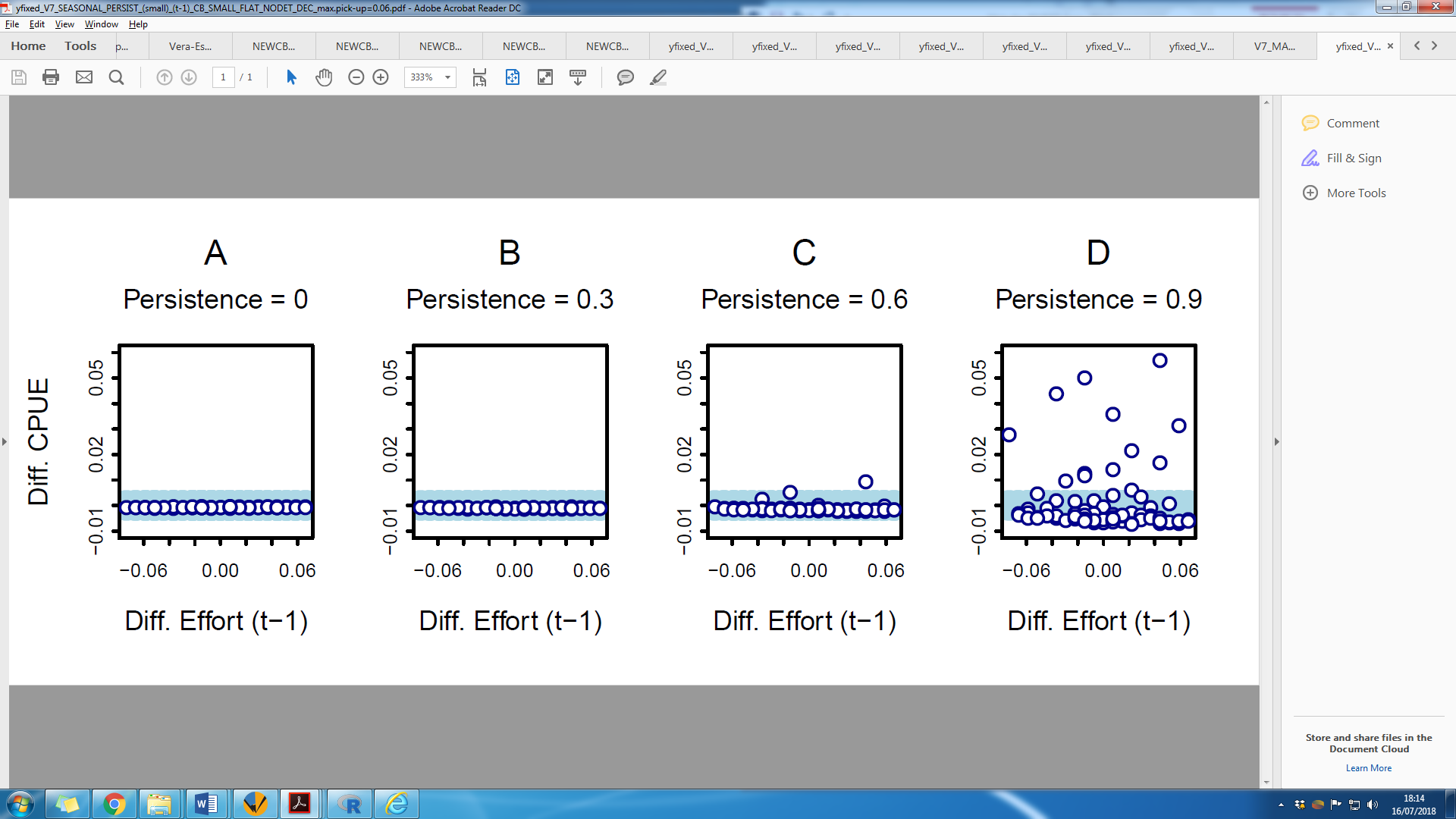


**Fig. S6.** Impact of persistence where there is no deterrence (as a contrast to Fig. 3). The scenario is exogenous decline, under the stable effort profile. All other scenarios with persistence are shown in Figs. S13-34.


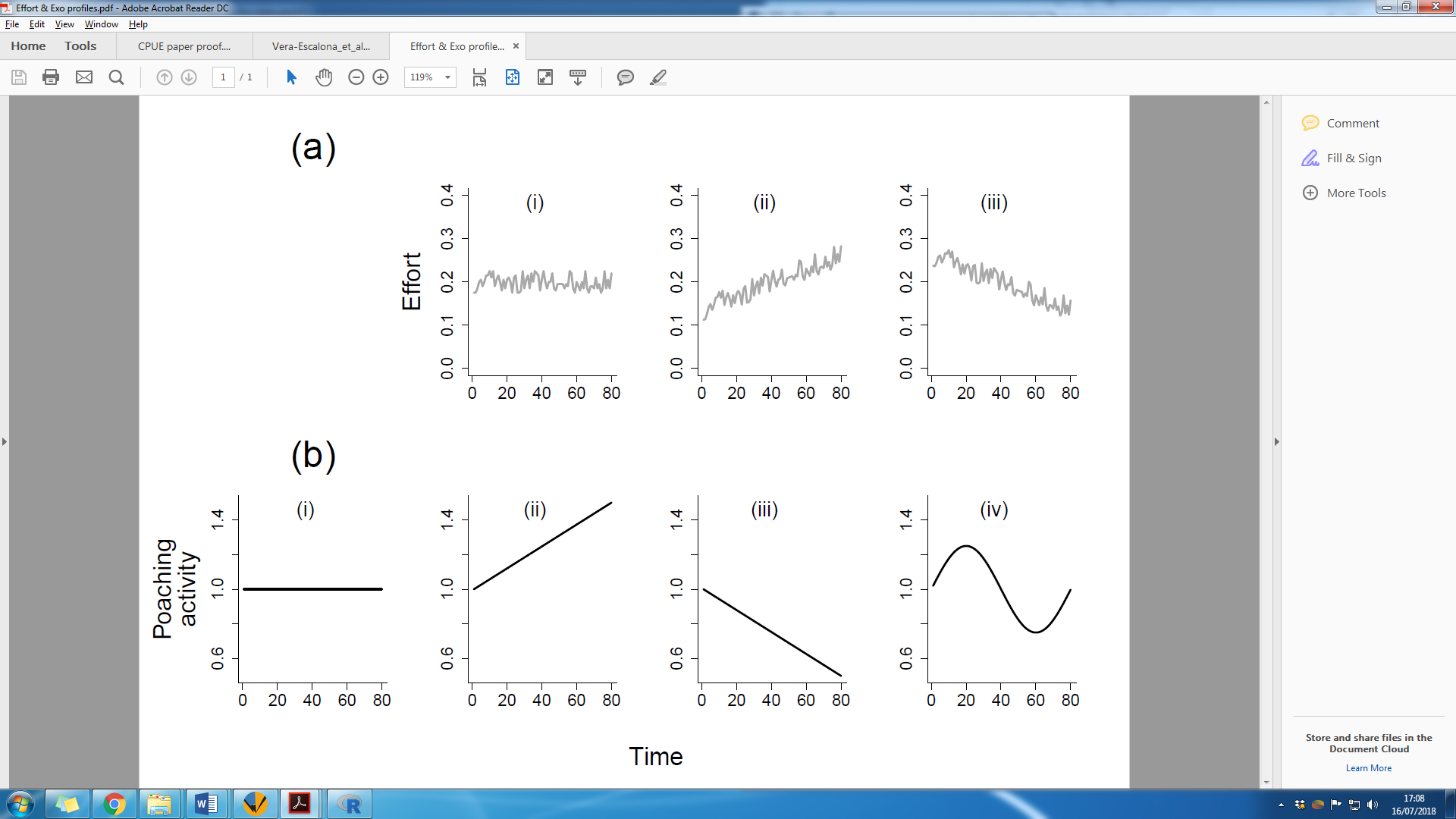


**Fig. S7.** The default effort and exogenous change profiles. (a) Effort profiles: (i) Decreasing, (ii), Stable, (iii) Increasing. ‘Noise’ added to the profiles is identical in each case. (b) Exogenous change profiles: (i) No change, (ii) Increasing, (iii) Decreasing, (iv) Seasonal.


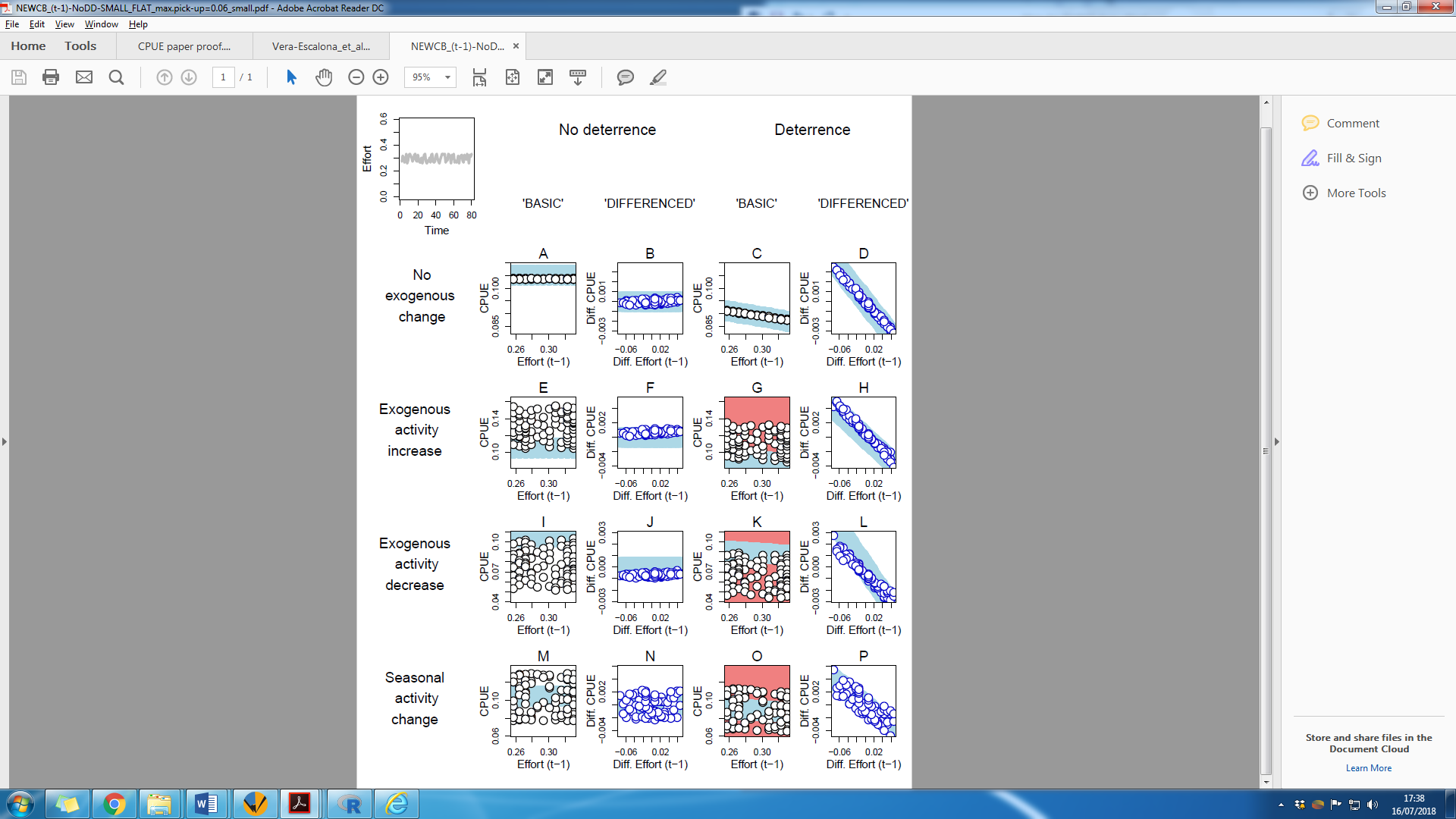


**Fig. S8.** Behavior of basic (black circles) and differenced (blue circles) CPUE-effort plots under a ‘stable’ effort profile in the six deterrence/exogenous-change scenarios (*n*=80 in each panel). See legend for Fig. 1 for more detail.


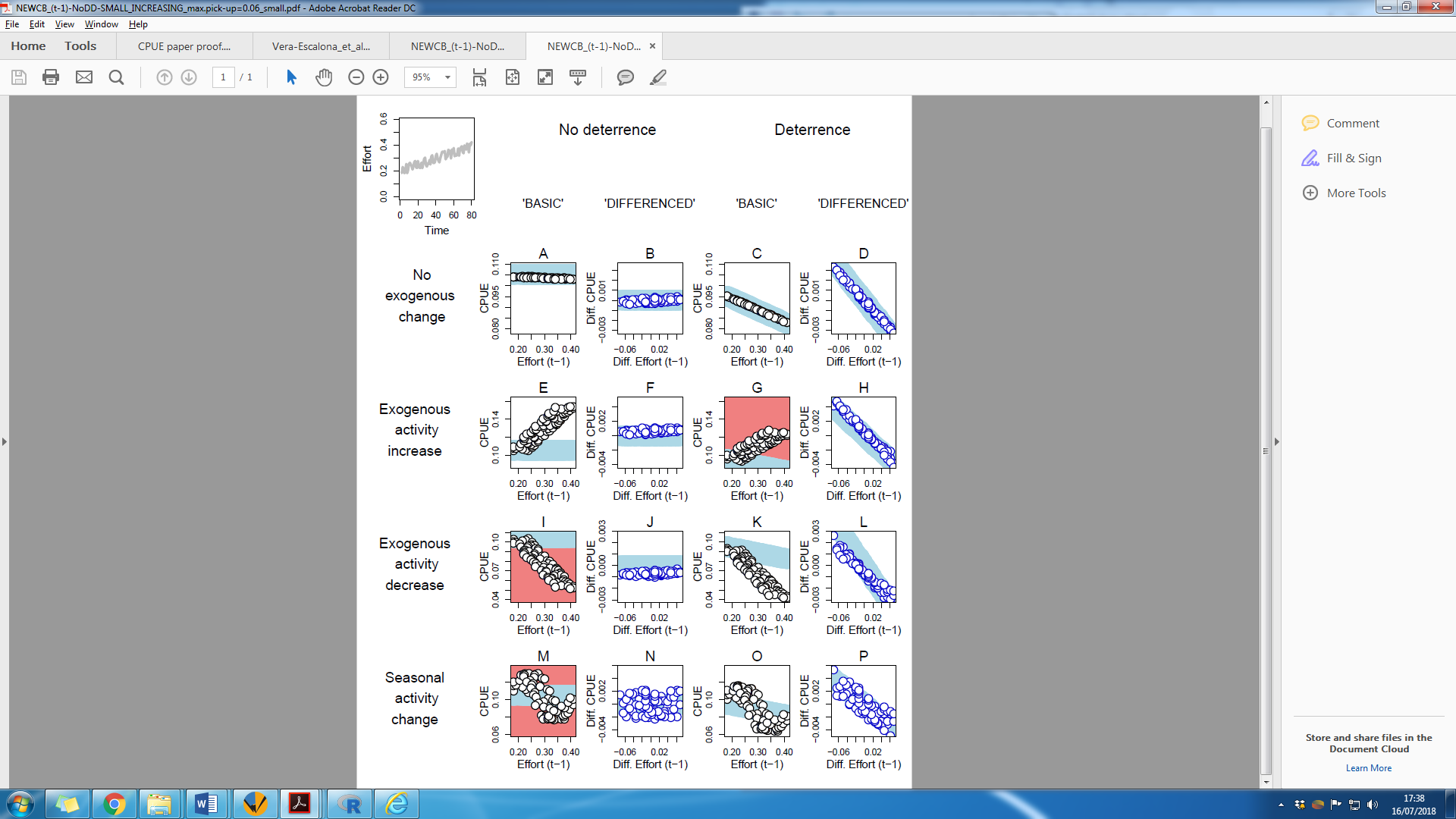


**Fig. S9.** Behavior of basic (black circles) and differenced (blue circles) CPUE-effort plots under an ‘increasing’ effort profile in the six deterrence/exogenous-change scenarios (*n*=80 in each panel). See legend for Fig. 1 for more detail.

**Figs S10-12.** Behavior of basic (black circles) and differenced (blue circles) CPUE-effort plots, with a moving average calculated over 5 timesteps. Plots are equivalent to Figs 1, S8 and S9.


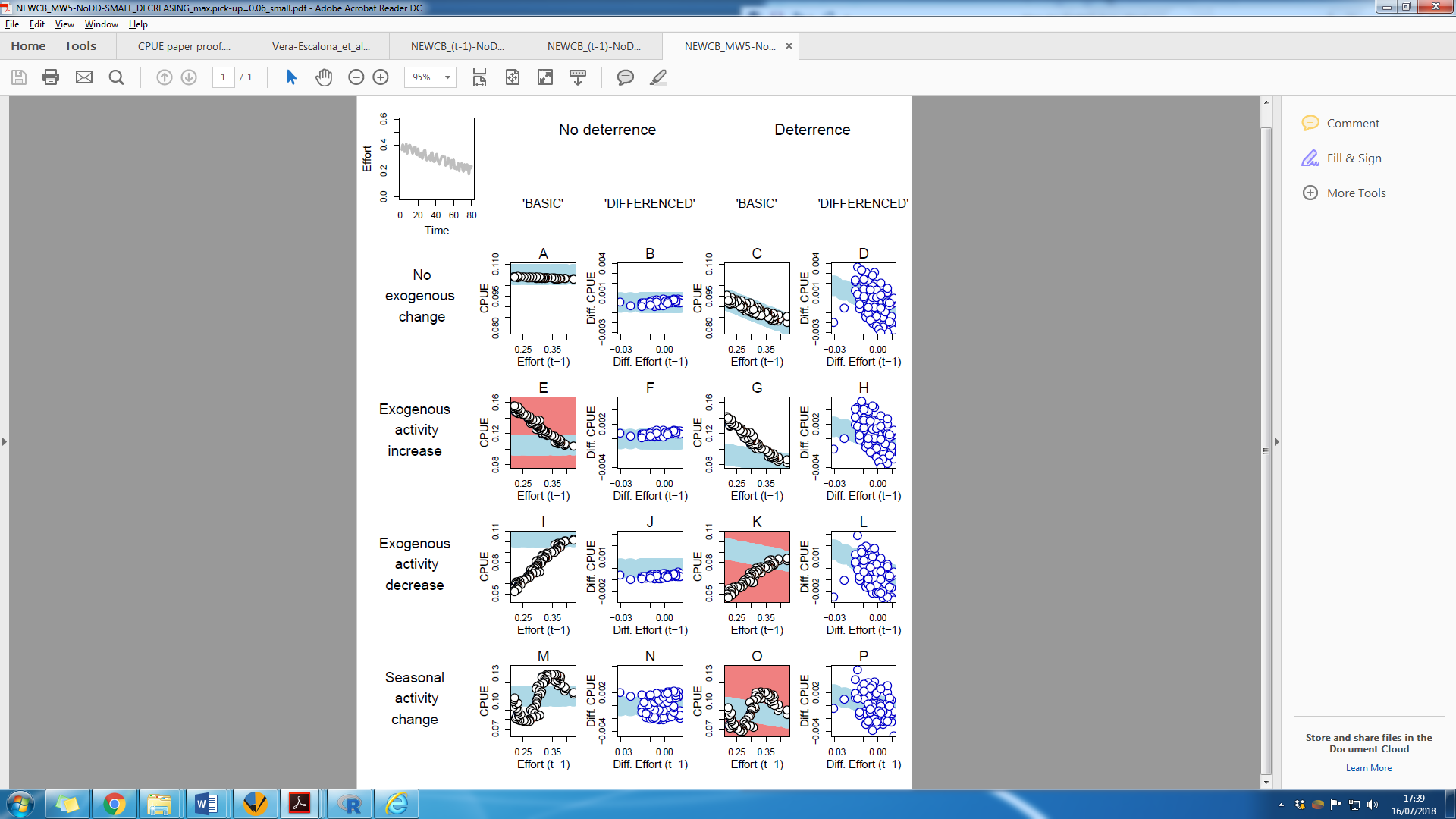


**Fig. S10.** MA plot, ‘decreasing’ effort profile.


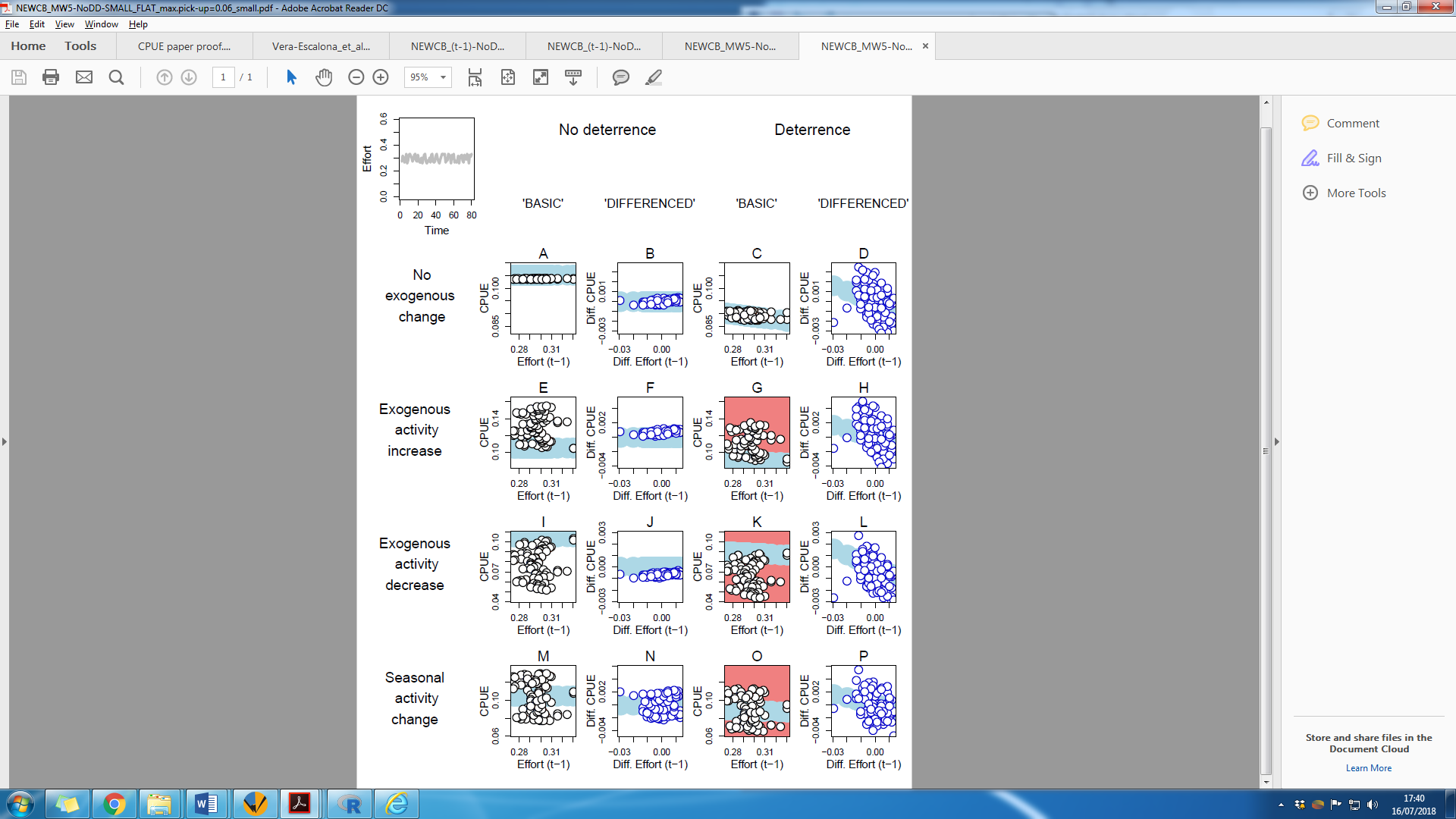


**Fig. S11.** MA plot, ‘stable’ effort profile.


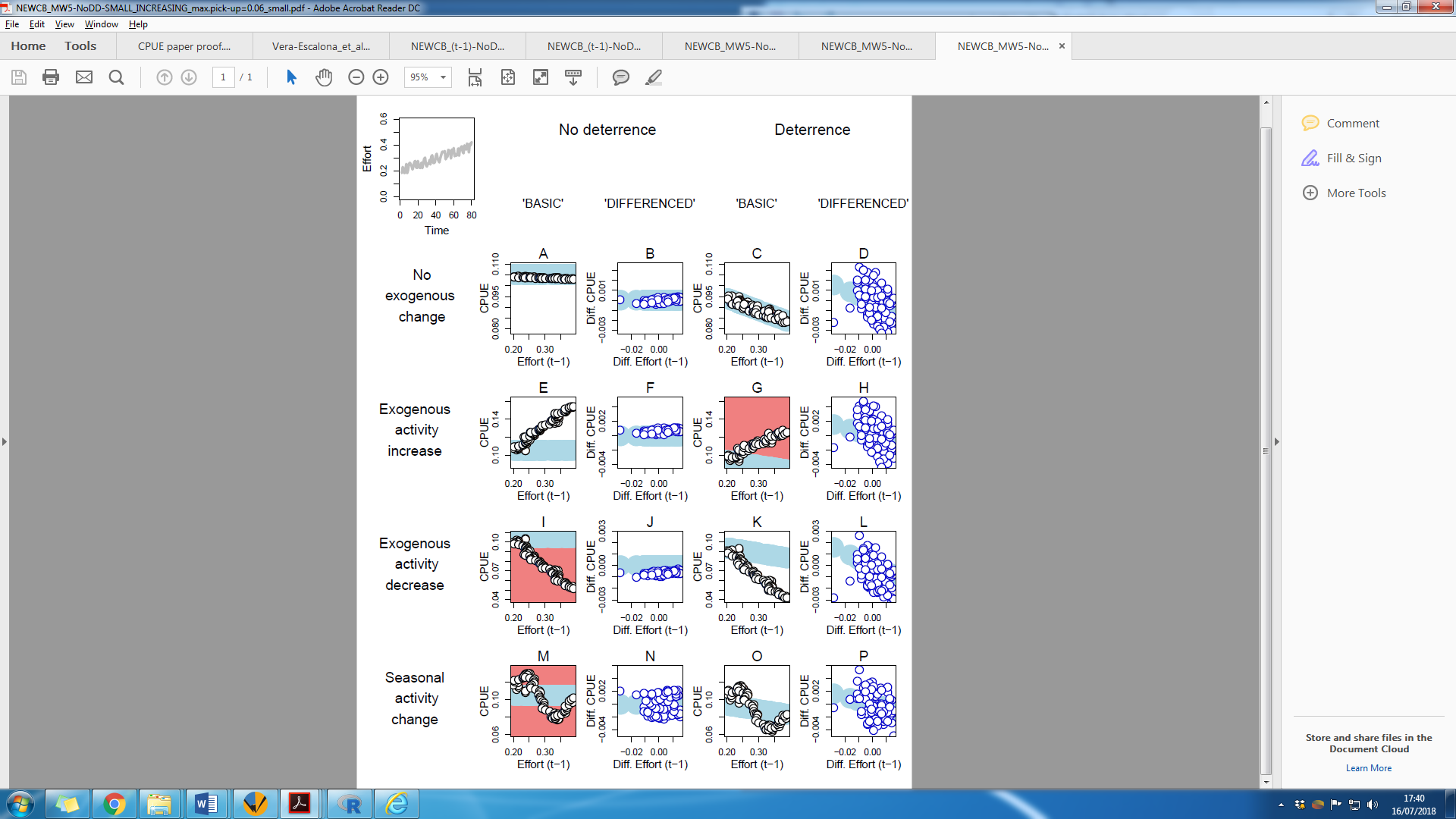


**Fig. S12.** MA plot, ‘increasing’ effort profile.

**Figs S13-23.** Impact of persistence on differenced CPUE-effort plots, with deterrence (*n*=80). These plots are equivalent to Fig. 3, but for all other combinations of exogenous change and effort profile.


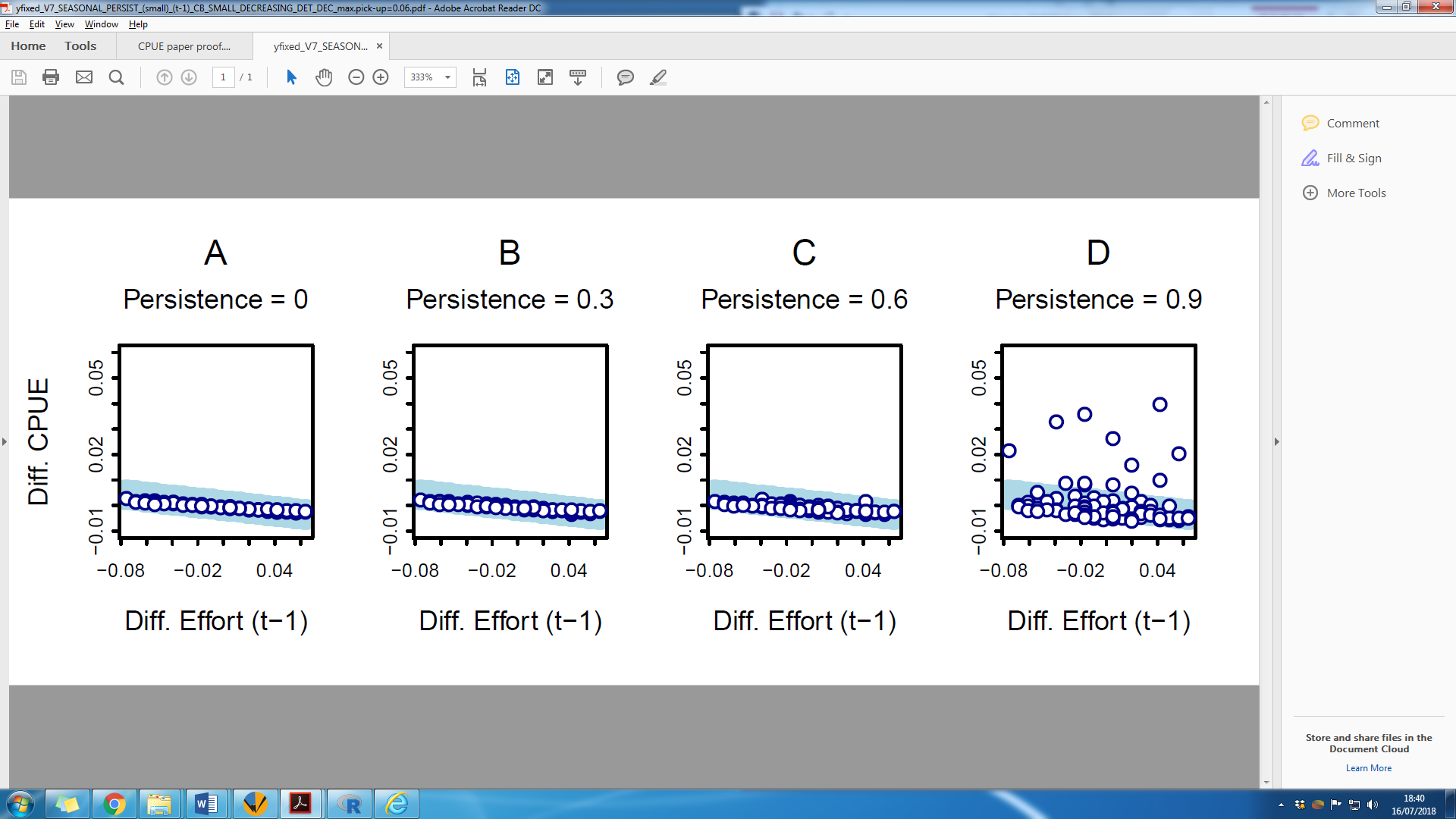


**Fig. S13.** Impact of persistence on differenced CPUE-effort plots (*n*=80). Decreasing effort profile, exogenous decline, with deterrence.


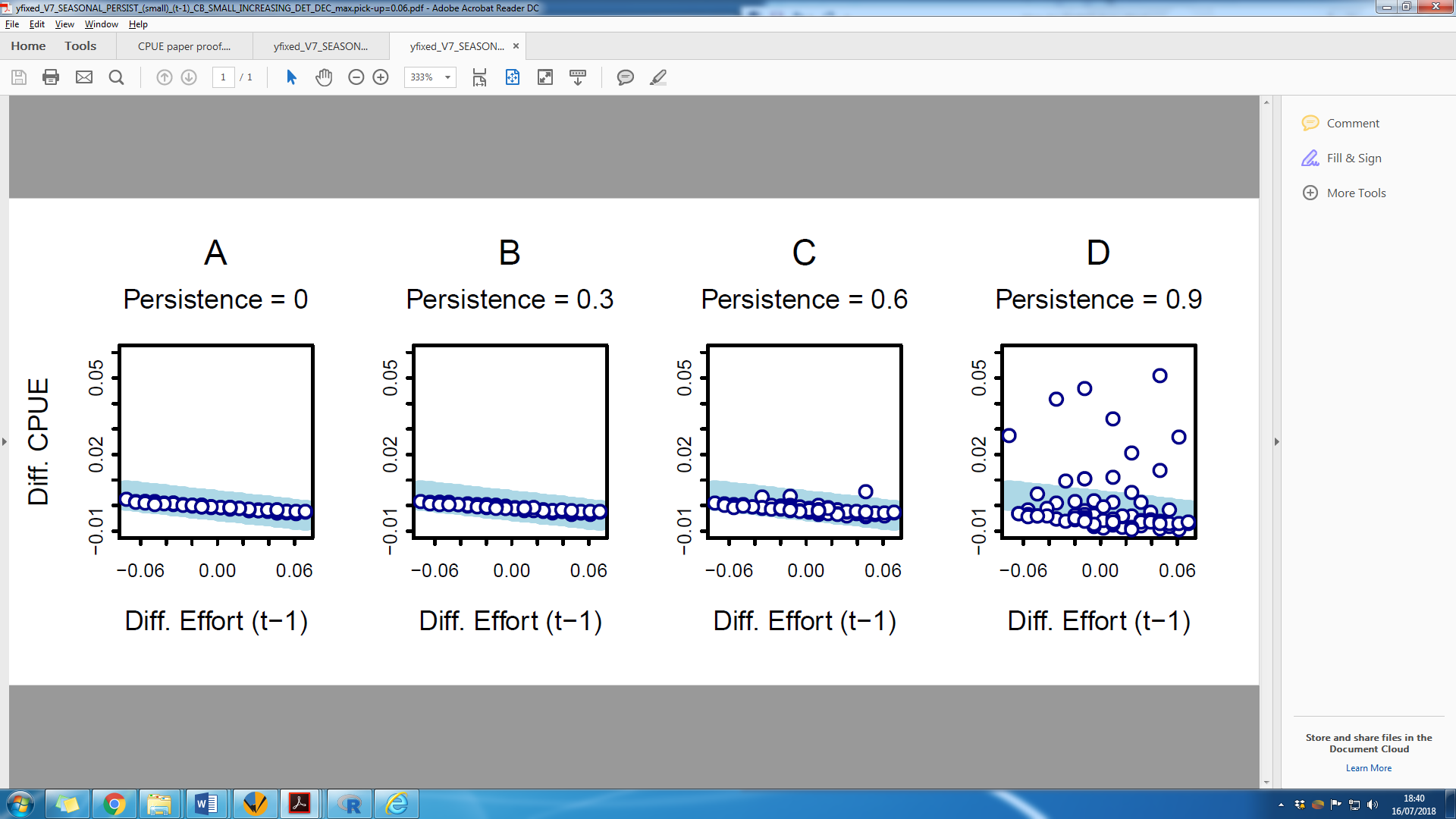


**Fig. S14.** Impact of persistence on differenced CPUE-effort plots (*n*=80). Increasing effort profile, exogenous decline, with deterrence.


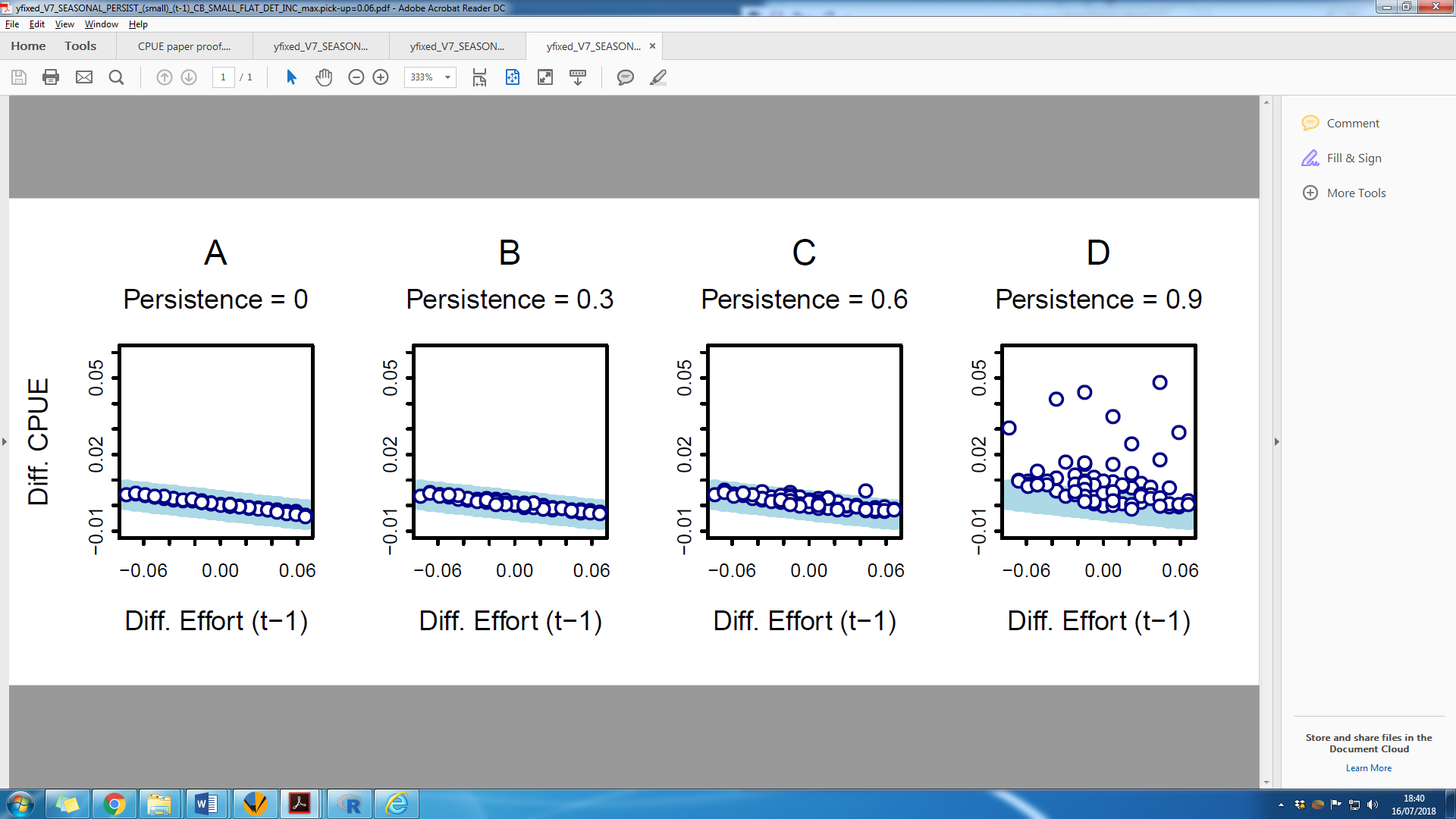


**Fig. S15.** Impact of persistence on differenced CPUE-effort plots (*n*=80). Stable effort profile, exogenous increase, with deterrence.


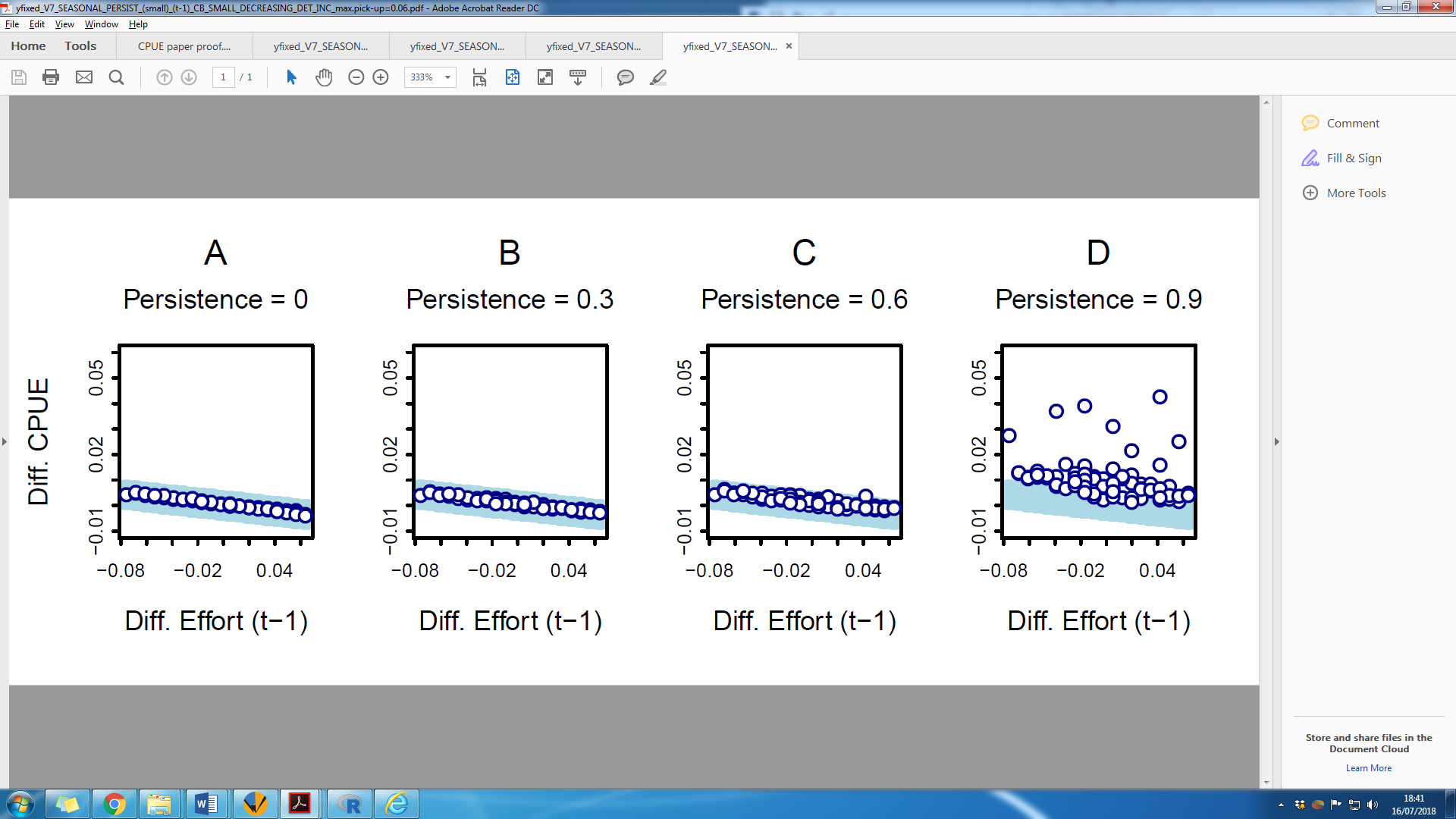


**Fig. S16.** Impact of persistence on differenced CPUE-effort plots (*n*=80). Decreasing effort profile, exogenous increase, with deterrence.


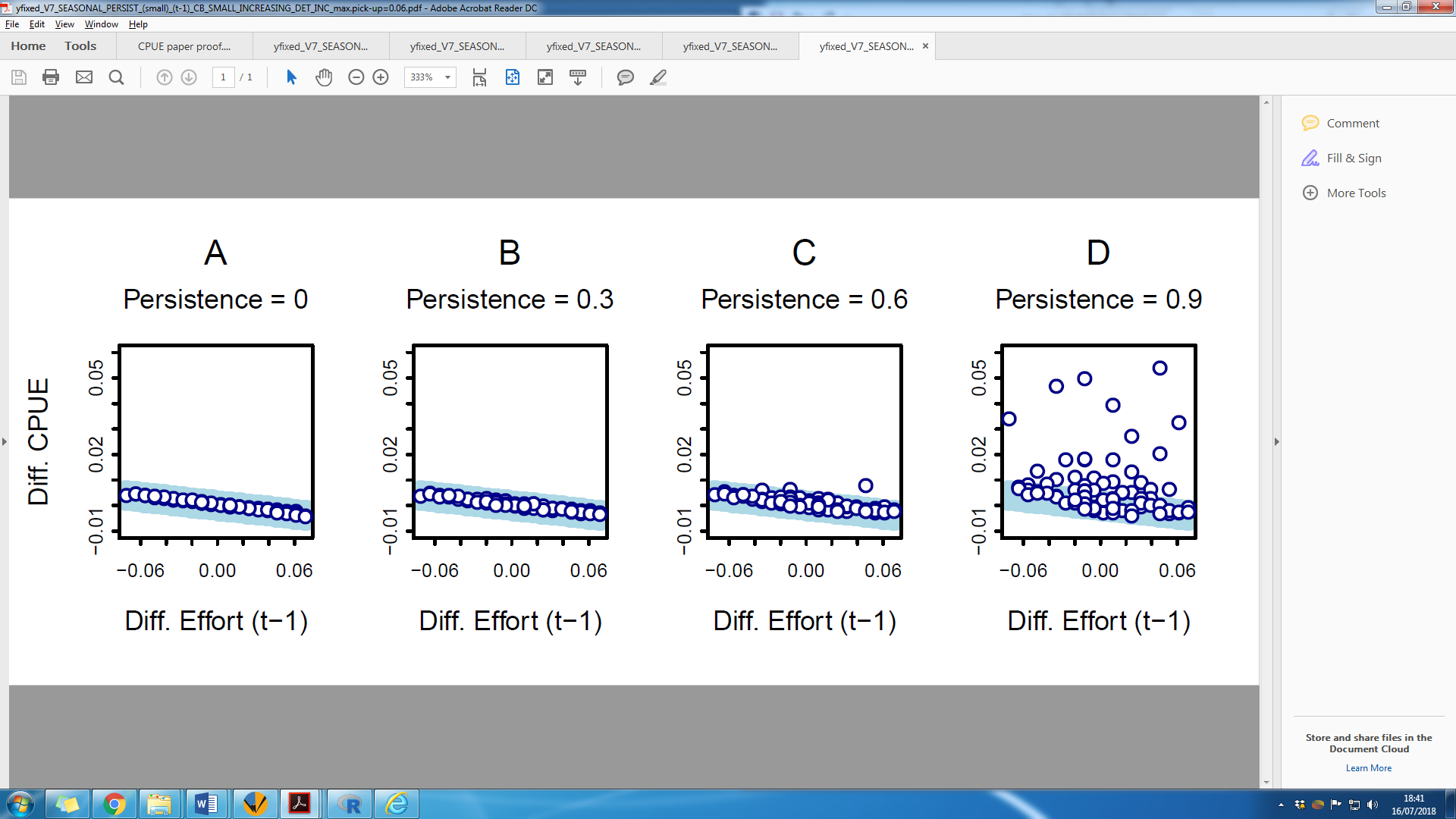
**Fig. S17.** Impact of persistence on differenced CPUE-effort plots (*n*=80). Increasing effort profile, exogenous increase, with deterrence.


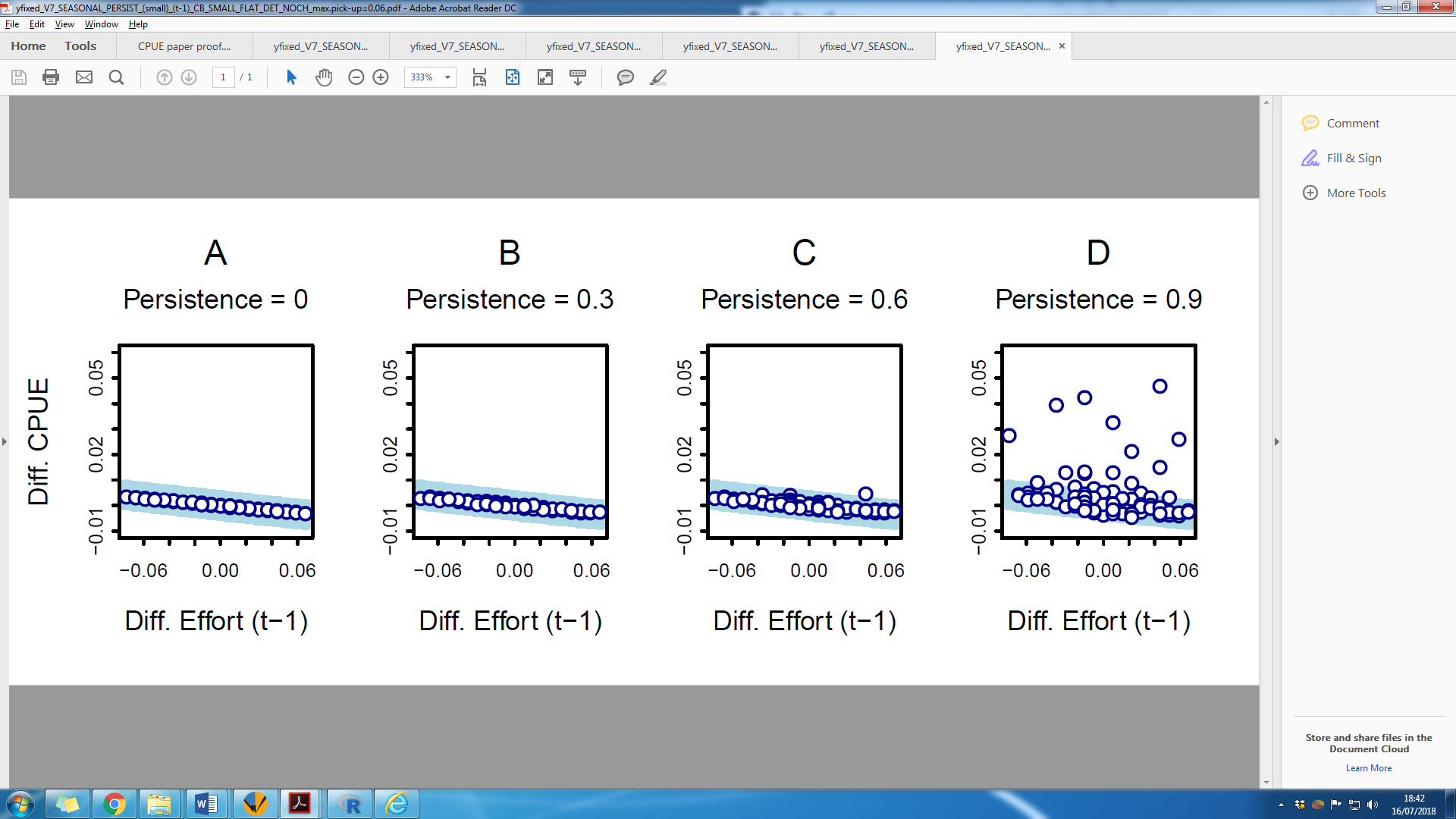


**Fig. S18.** Impact of persistence on differenced CPUE-effort plots (*n*=80). Stable effort profile, no exogenous change, with deterrence.


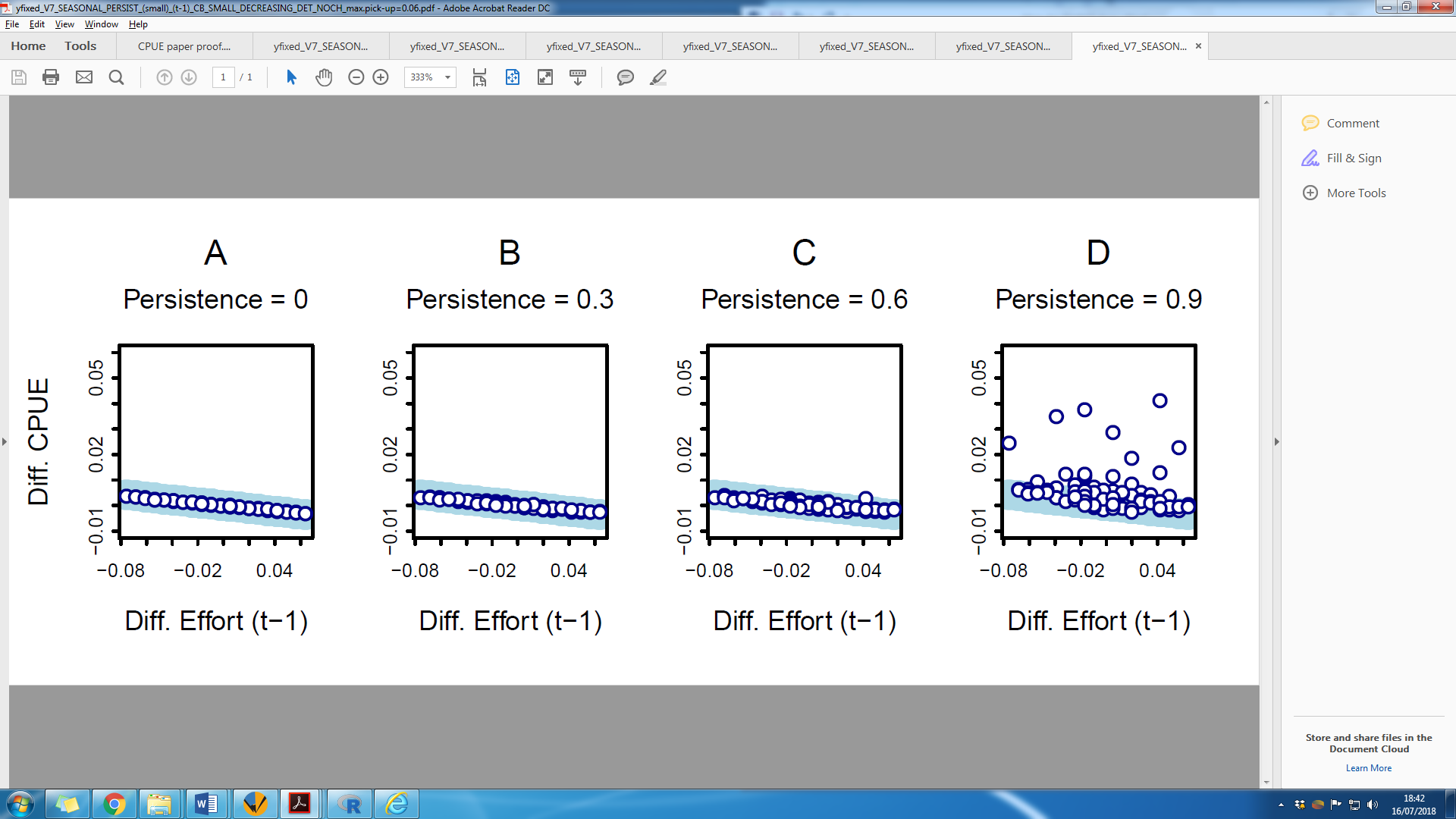


**Fig. S19.** Impact of persistence on differenced CPUE-effort plots (*n*=80). Decreasing effort profile, no exogenous change, with deterrence.


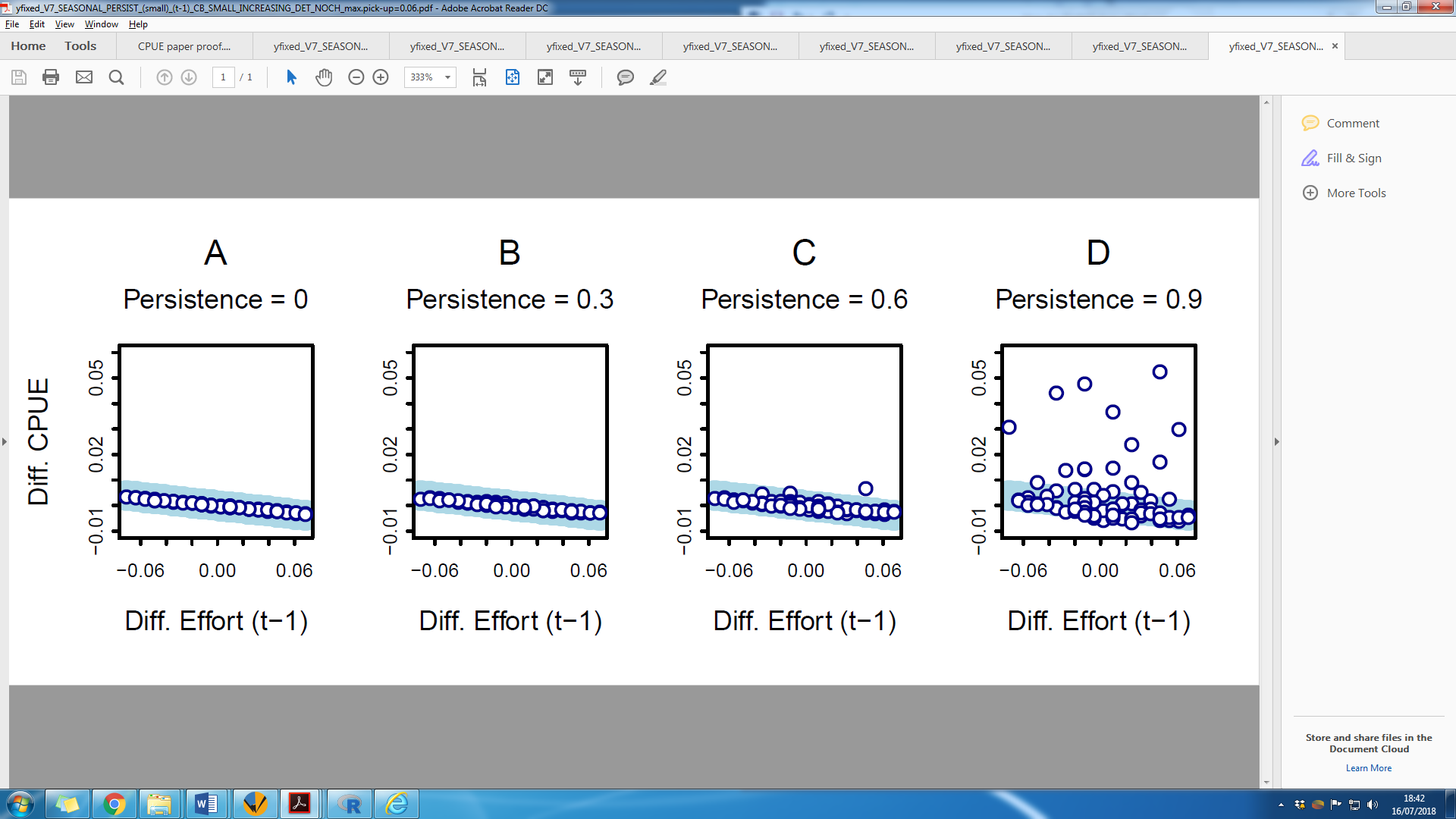


**Fig. S20.** Impact of persistence on differenced CPUE-effort plots (*n*=80). Increasing effort profile, no exogenous change, with deterrence.


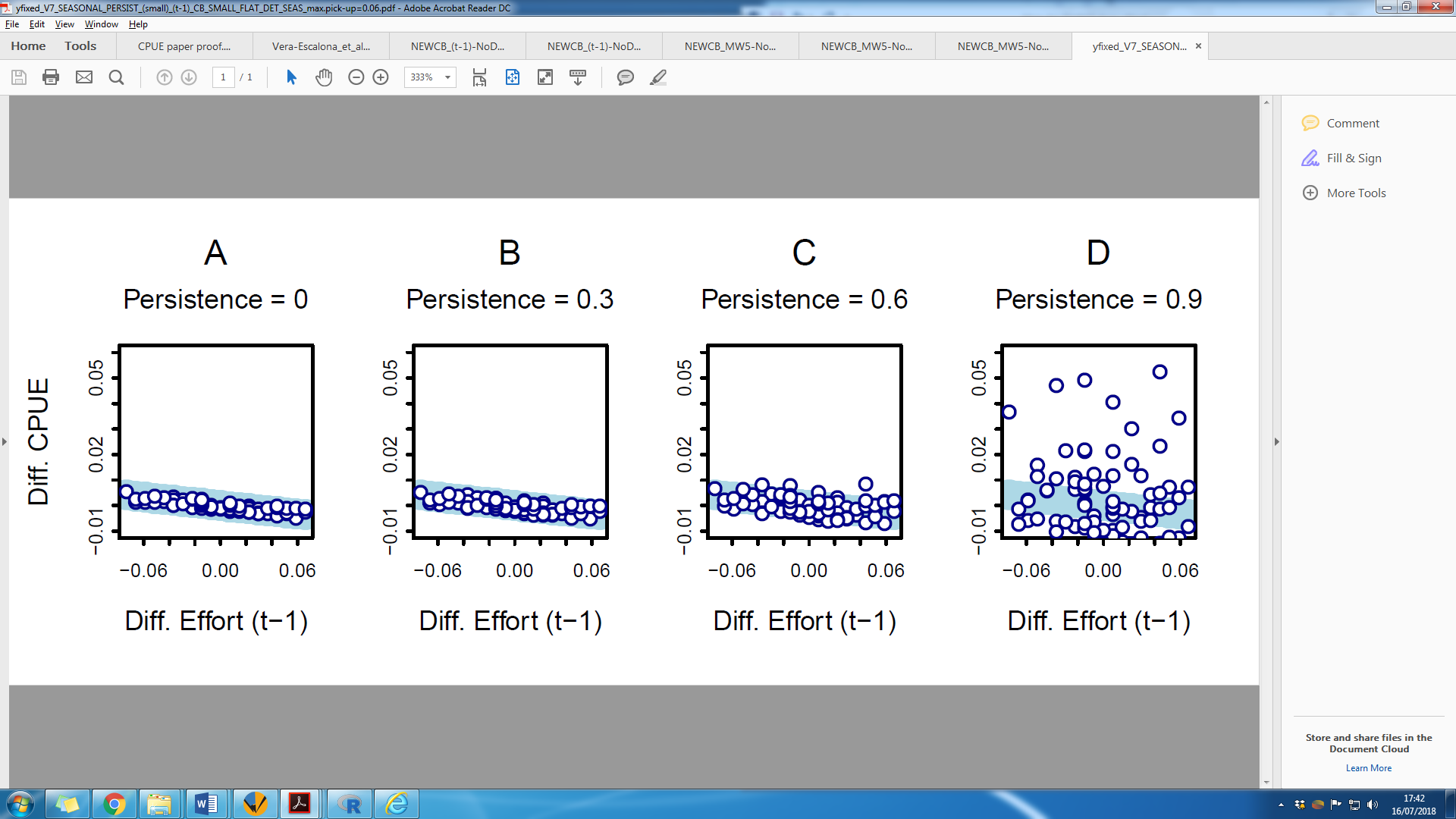


**Fig. S21.** Impact of persistence on differenced CPUE-effort plots (*n*=80). Stable effort profile, seasonal exogenous change, with deterrence.


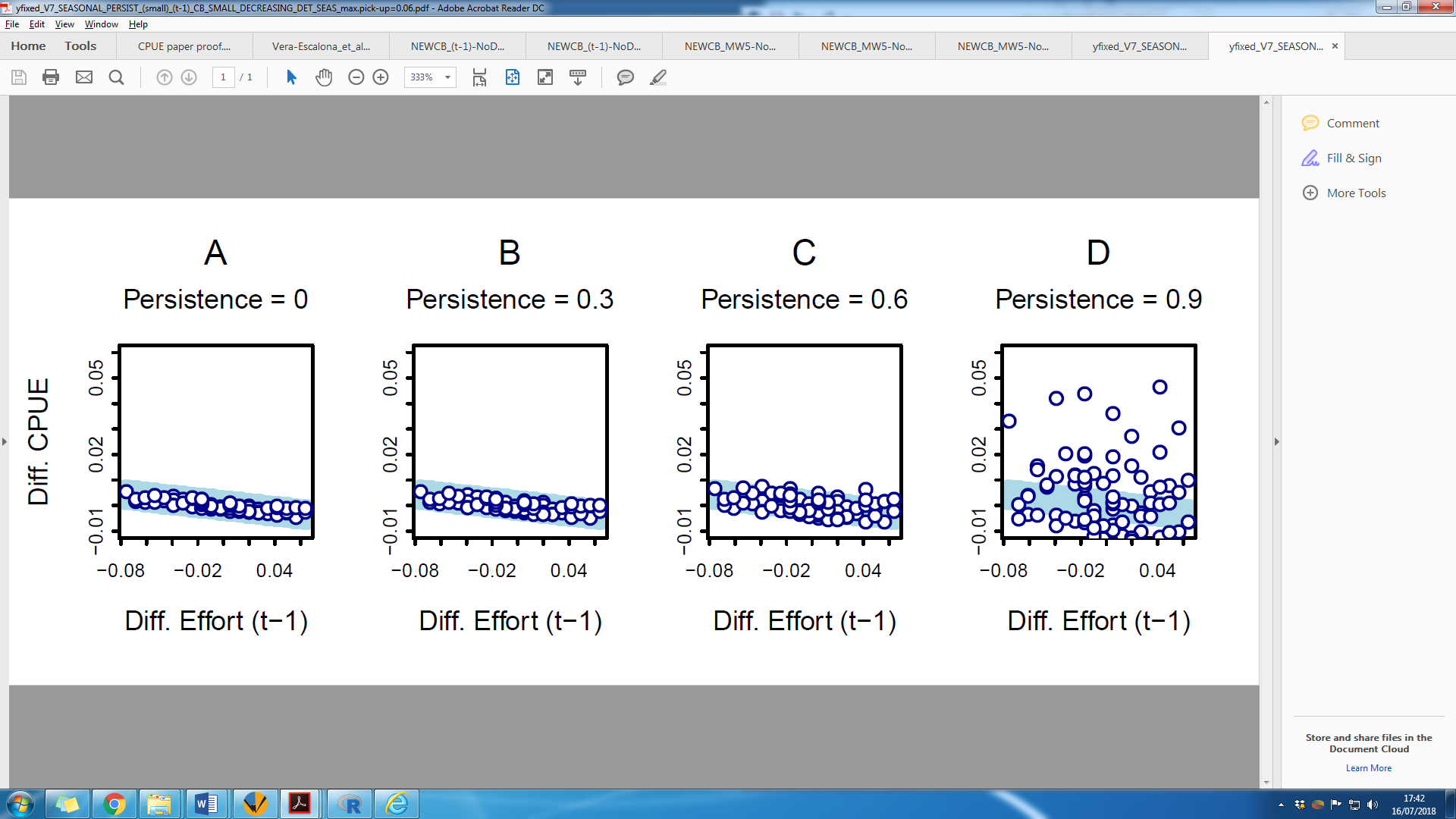


**Fig. S22.** Impact of persistence on differenced CPUE-effort plots (*n*=80). Decreasing effort profile, seasonal exogenous change, with deterrence.


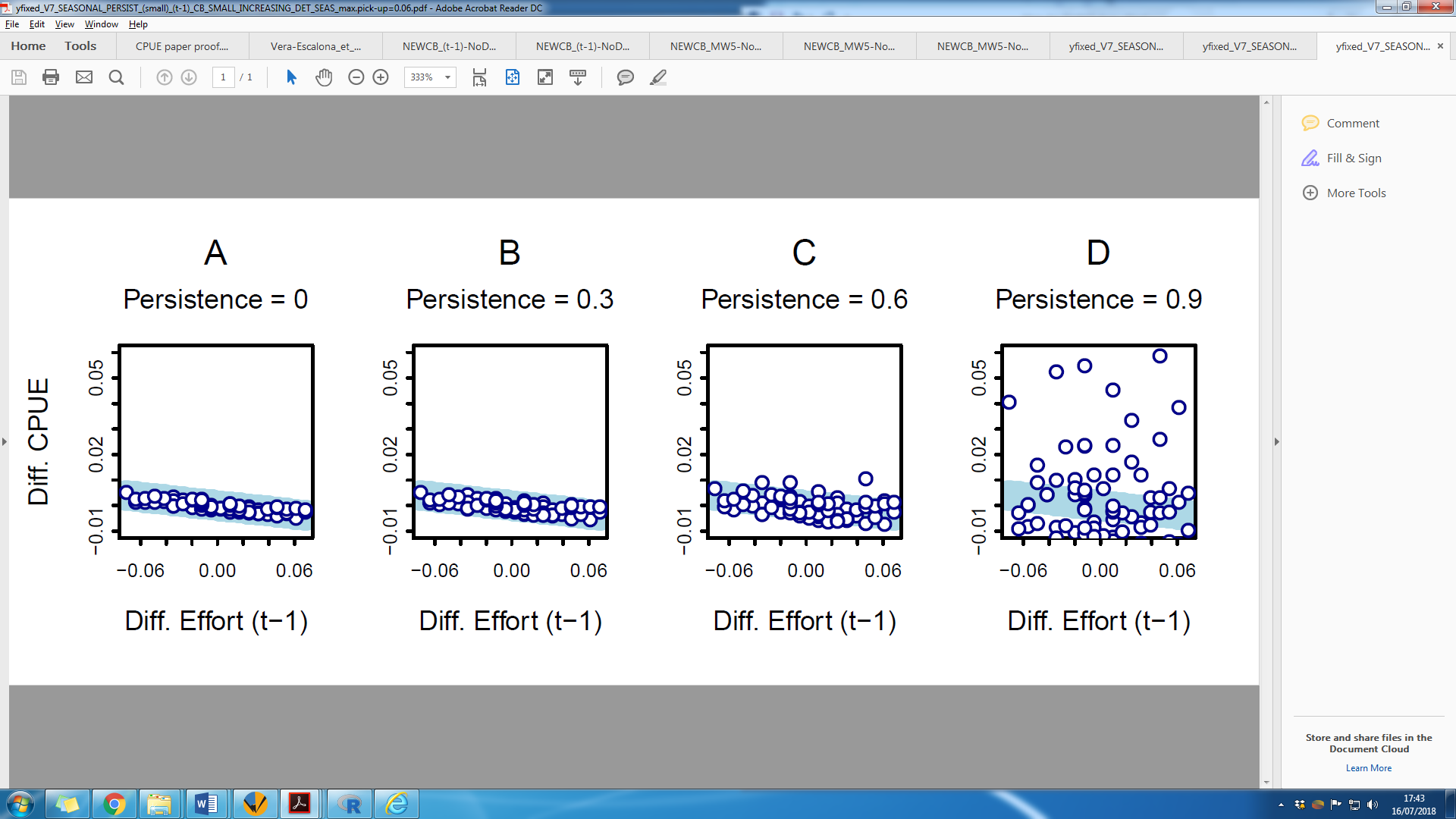


**Fig. S23.** Impact of persistence on differenced CPUE-effort plots (*n*=80). Increasing effort profile, seasonal exogenous change, with deterrence.

**Figs S24-32.** Impact of persistence on differenced CPUE-effort plots, with no deterrence (*n*=80). These plots are equivalent to Fig. S6, but for all other combinations of exogenous change and effort profile. (Plots are very similar).


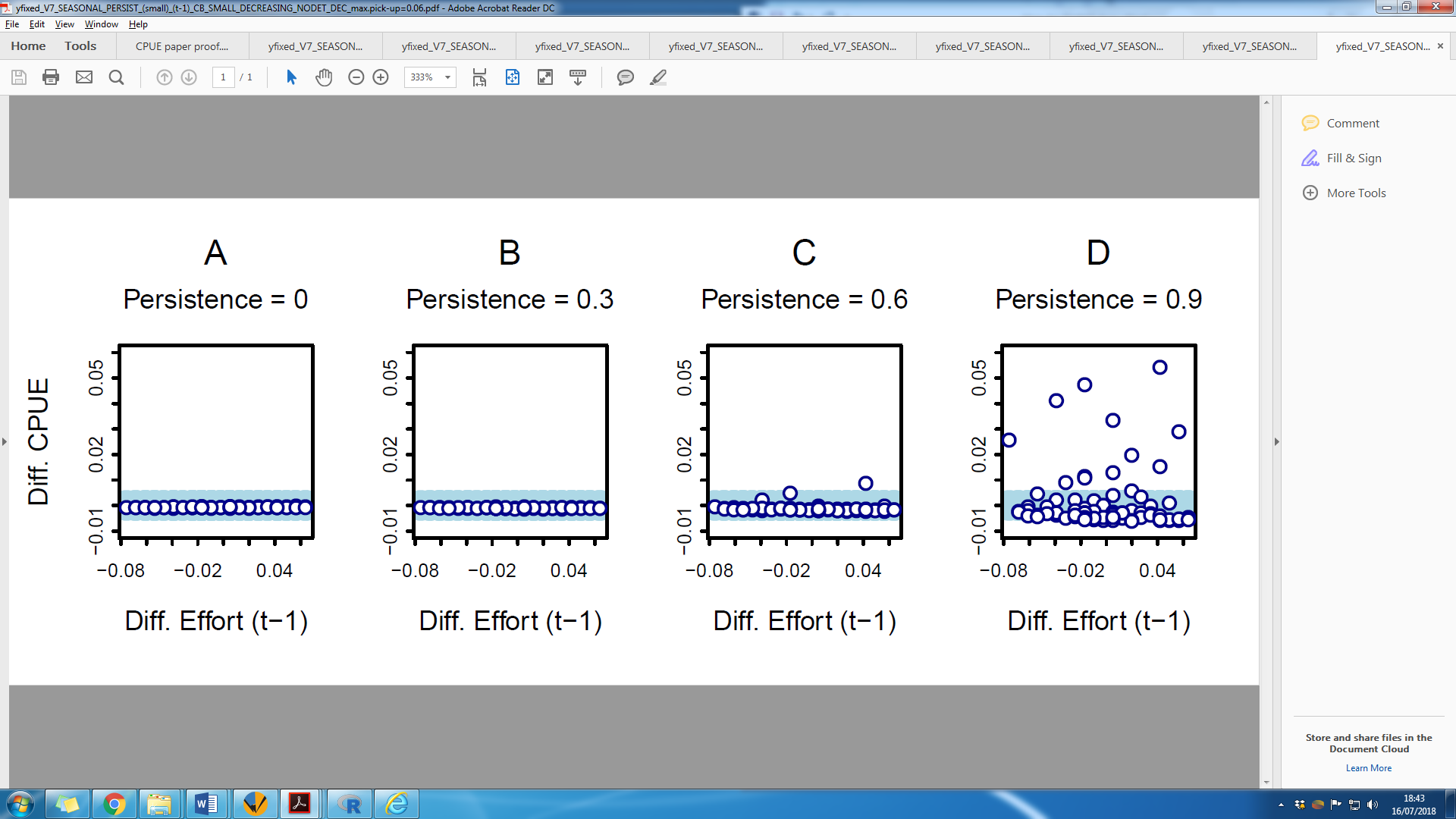


**Fig. S24.** Impact of persistence on differenced CPUE-effort plots (*n*=80). Decreasing effort profile, exogenous decline, no deterrence.


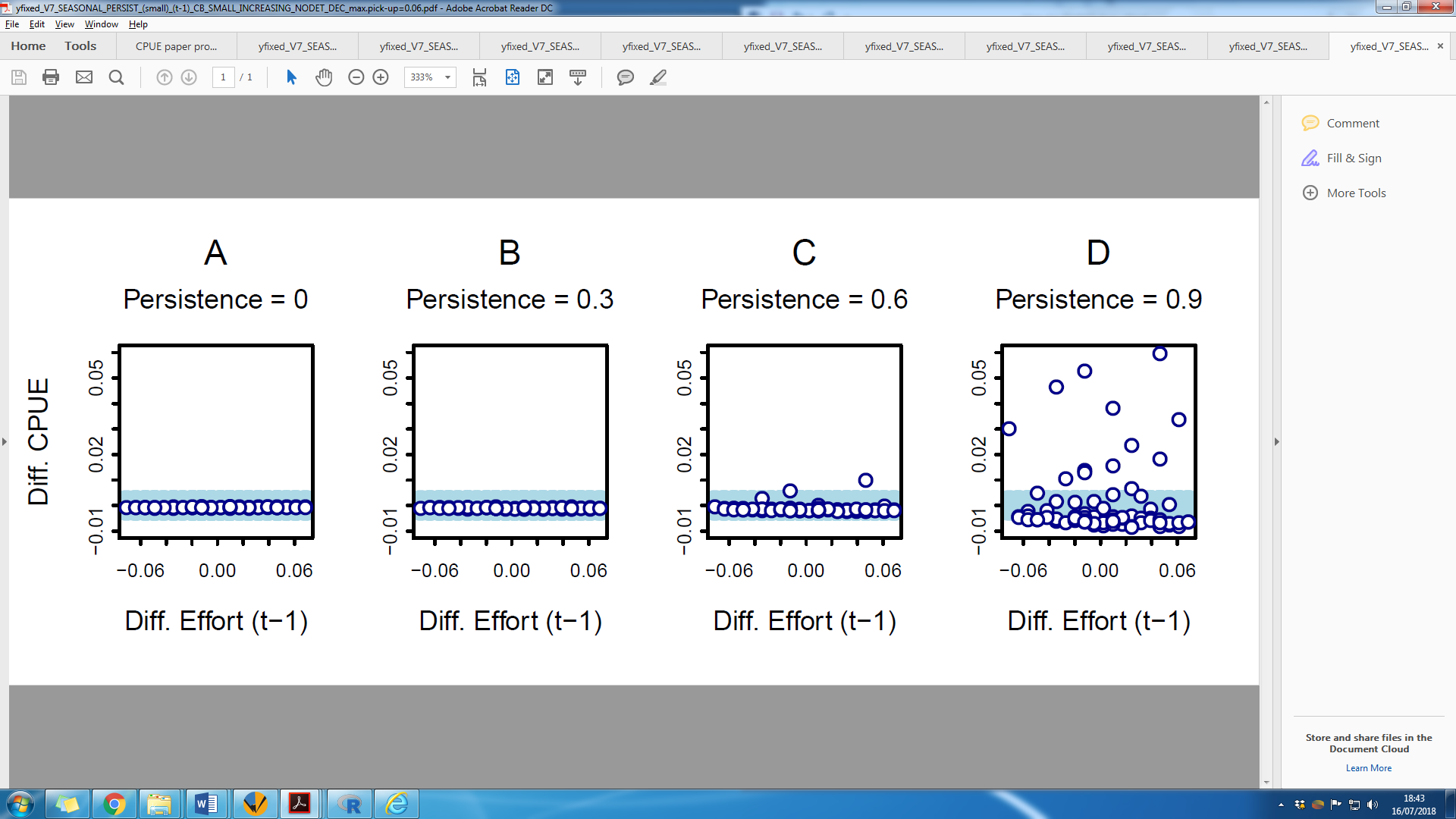


**Fig. S25.** Impact of persistence on differenced CPUE-effort plots (*n*=80). Increasing effort profile, exogenous decline, no deterrence.


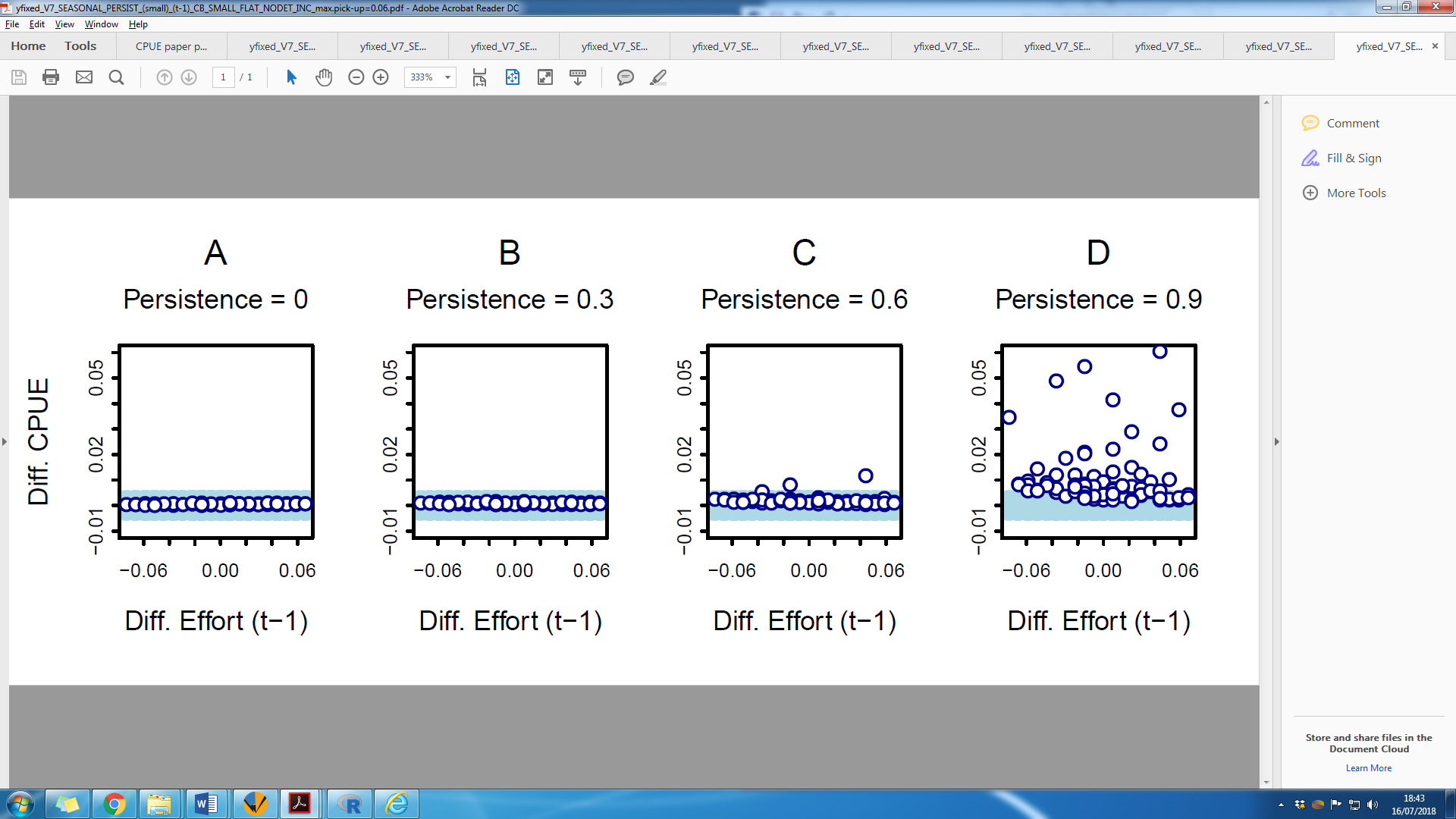


**Fig. S26.** Impact of persistence on differenced CPUE-effort plots (*n*=80). Stable effort profile, exogenous increase, no deterrence.


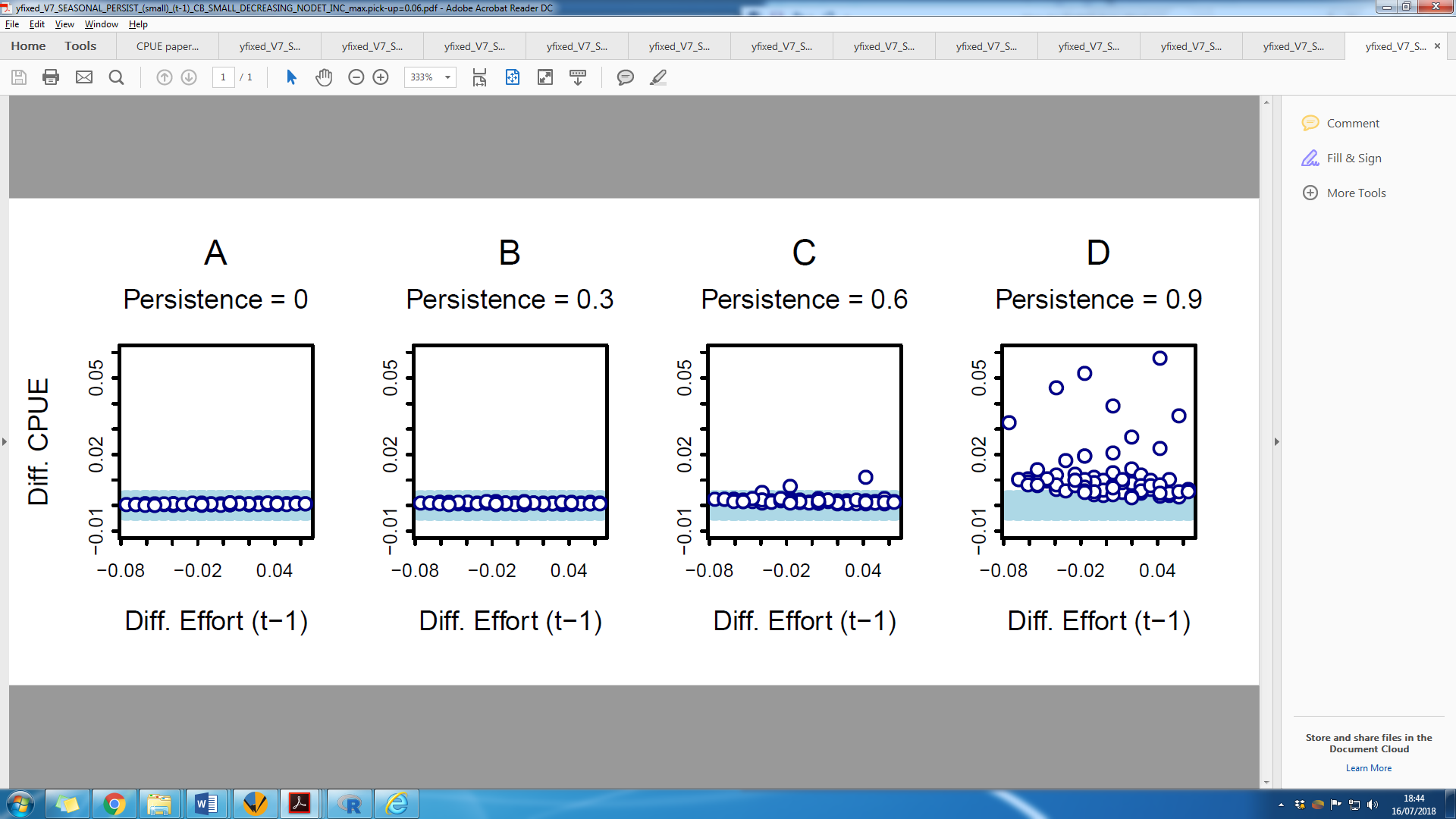


**Fig. S27.** Impact of persistence on differenced CPUE-effort plots (*n*=80). Decreasing effort profile, exogenous increase, no deterrence.


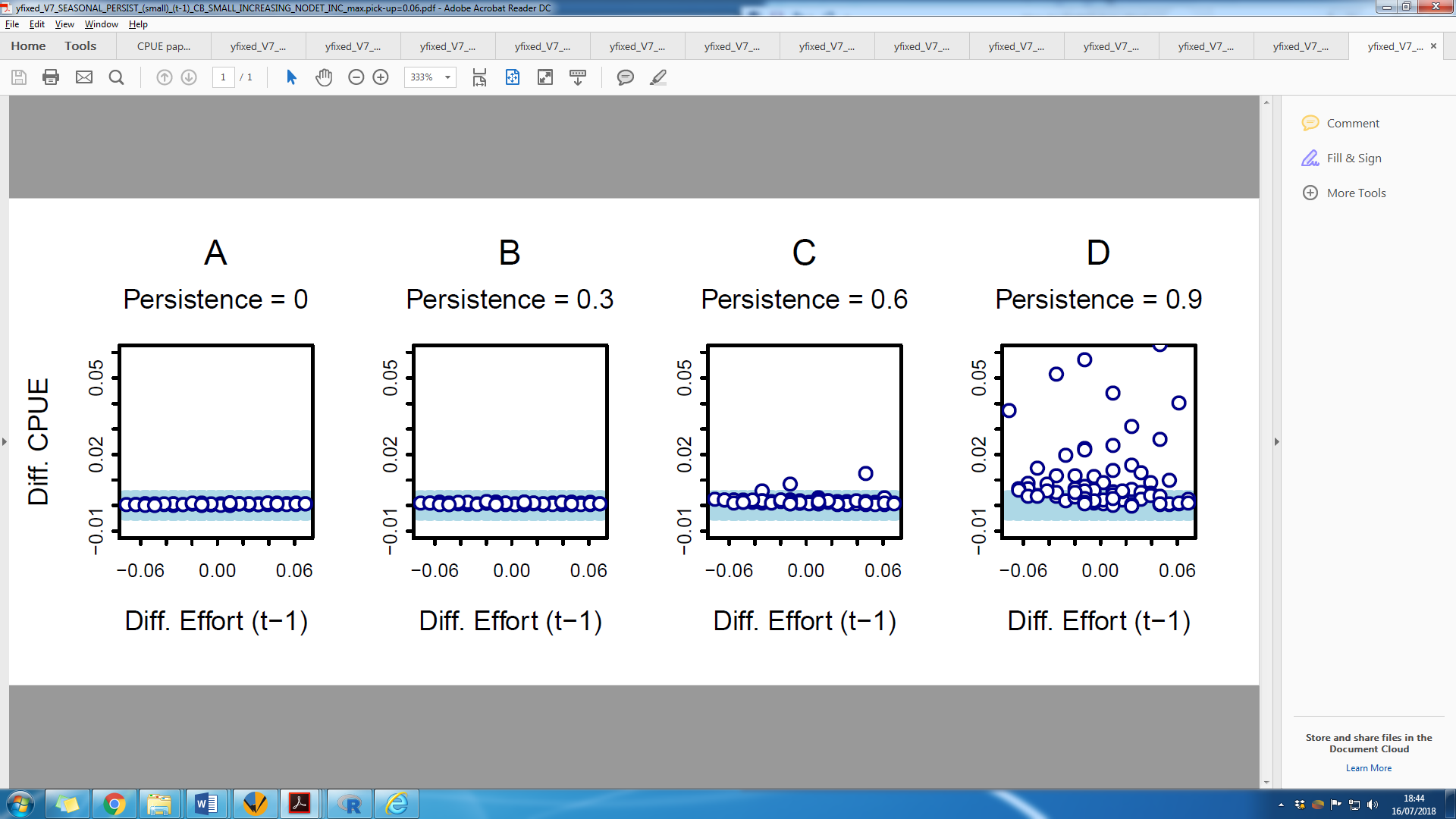


**Fig. S28.** Impact of persistence on differenced CPUE-effort plots (*n*=80). Increasing effort profile, exogenous increase, no deterrence.


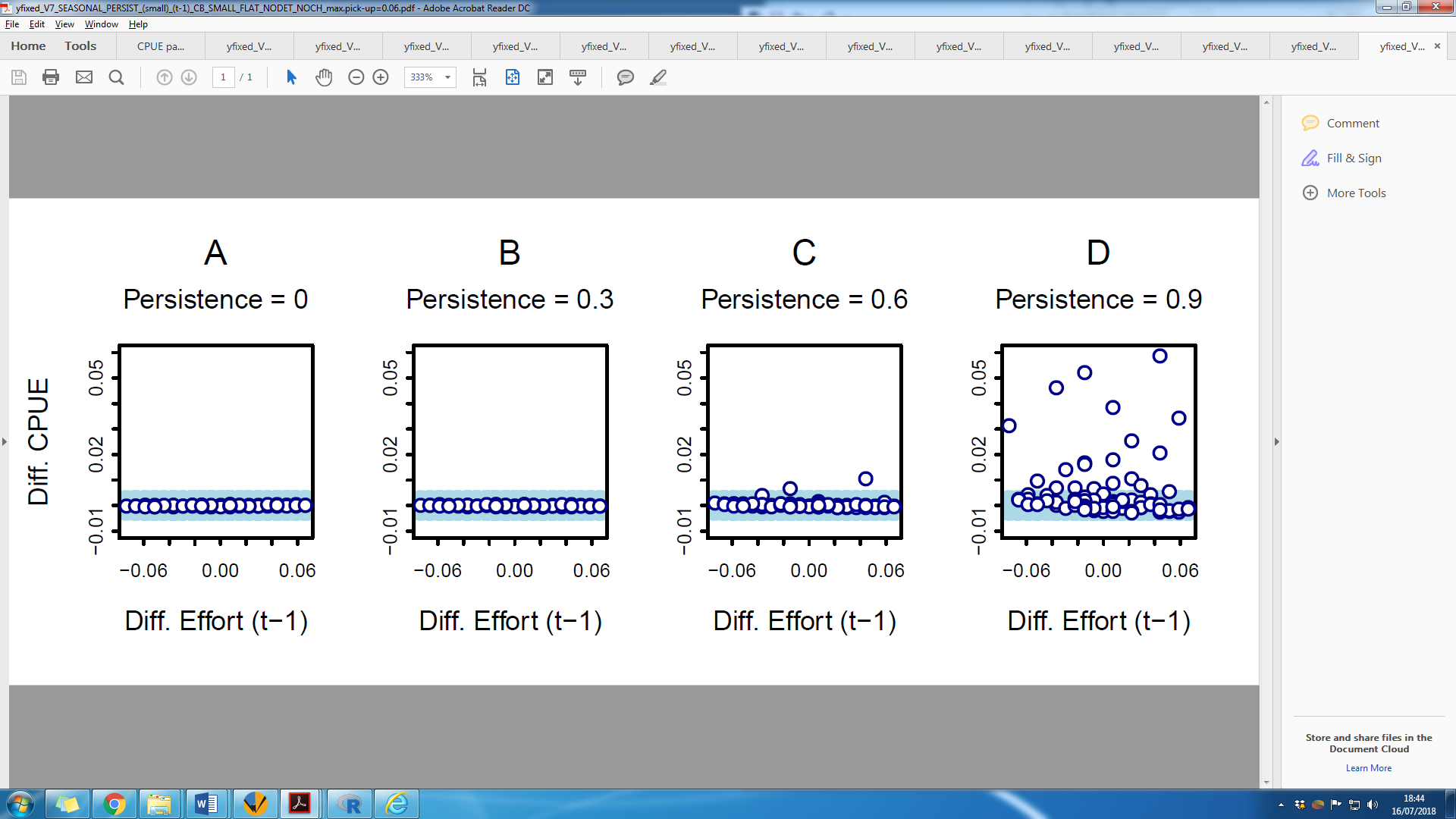


**Fig. S29.** Impact of persistence on differenced CPUE-effort plots (*n*=80). Stable effort profile, no exogenous change, no deterrence.


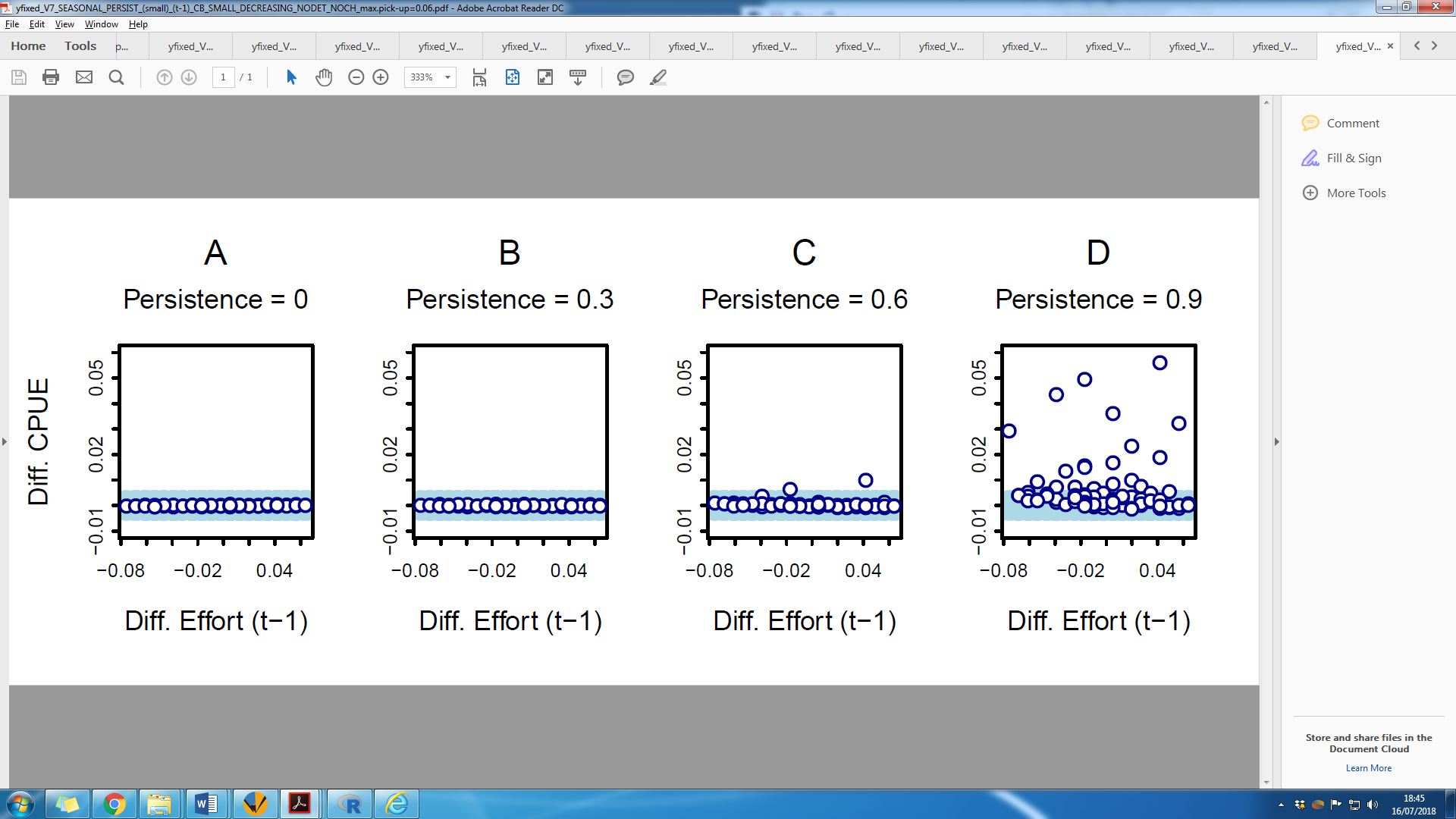


**Fig. S30.** Impact of persistence on differenced CPUE-effort plots (*n*=80). Decreasing effort profile, no exogenous change, no deterrence.


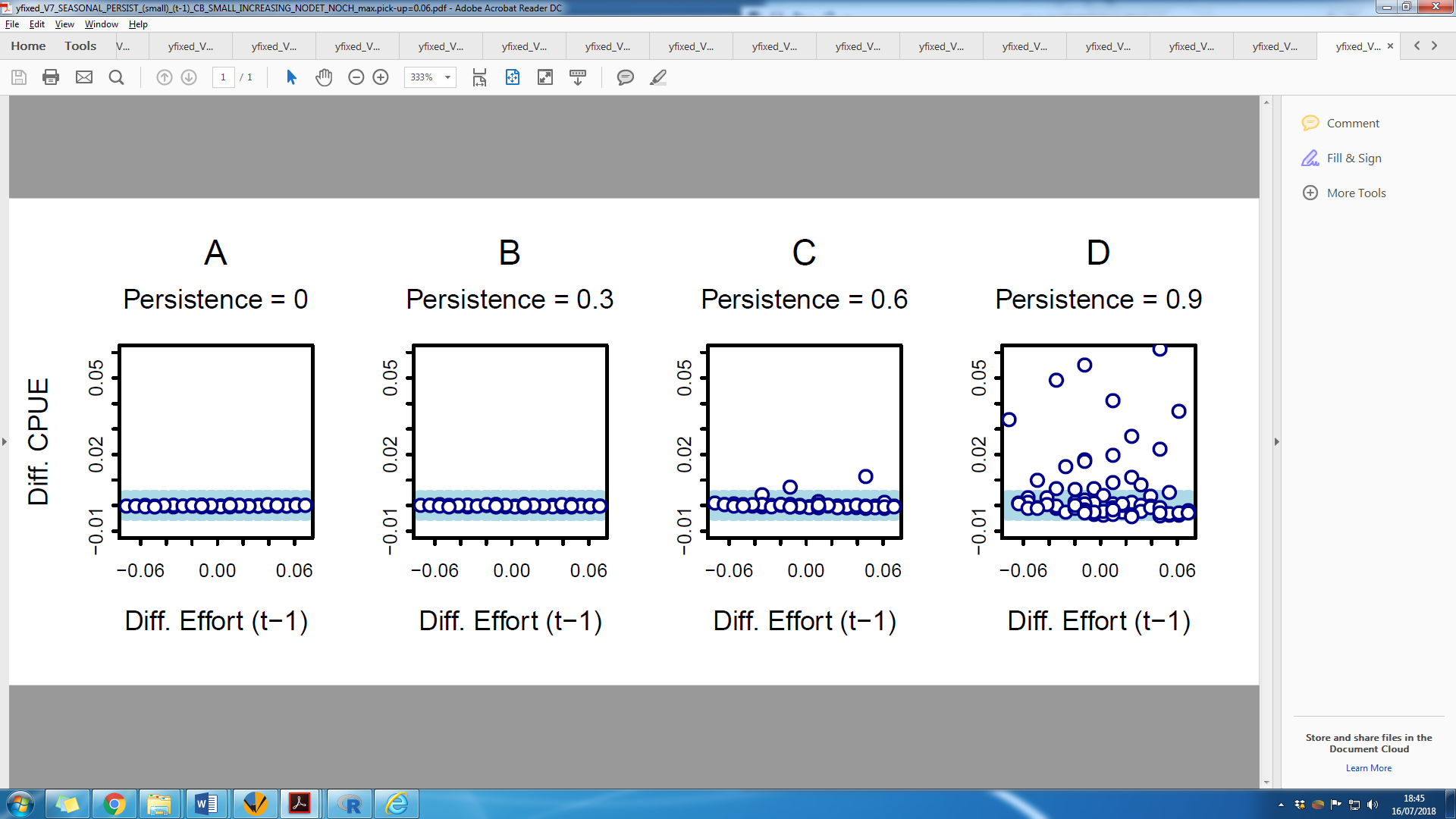


**Fig. S31.** Impact of persistence on differenced CPUE-effort plots (*n*=80). Increasing effort profile, no exogenous change, no deterrence.


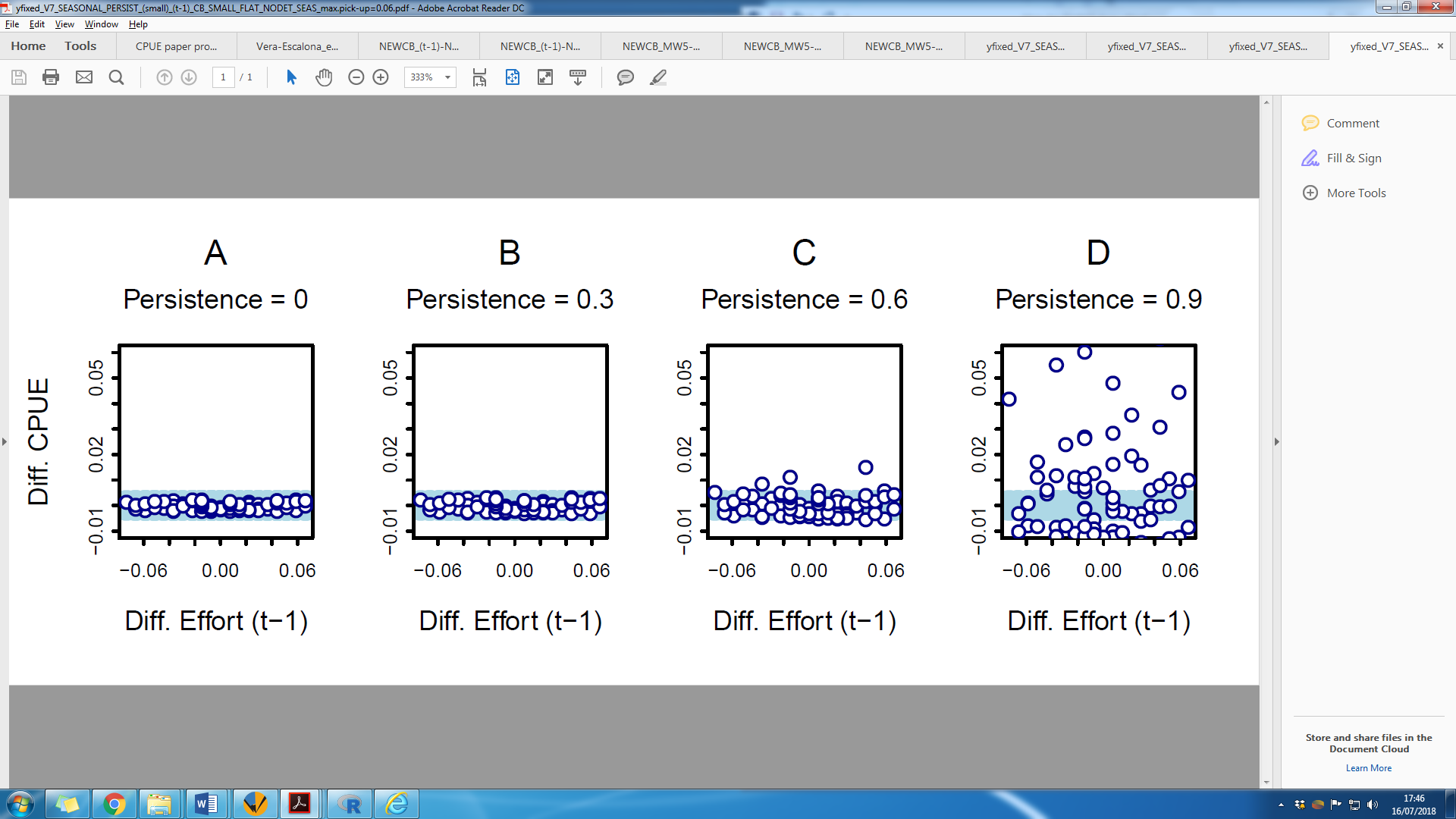


**Fig. S32.** Impact of persistence on differenced CPUE-effort plots (*n*=80). Stable effort profile, seasonal exogenous change, no deterrence.


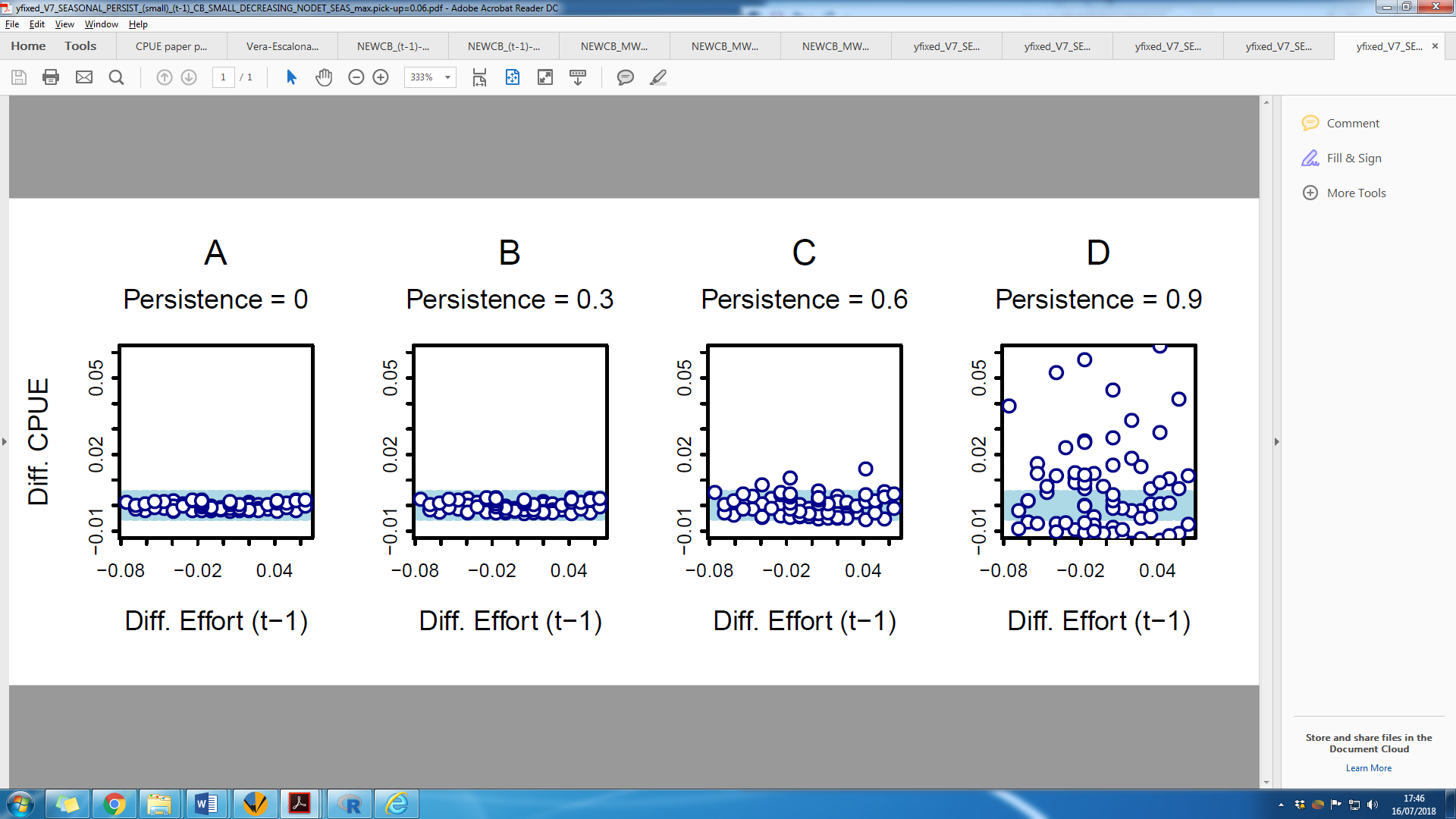


**Fig. S33.** Impact of persistence on differenced CPUE-effort plots (*n*=80). Decreasing effort profile, seasonal exogenous change, no deterrence.


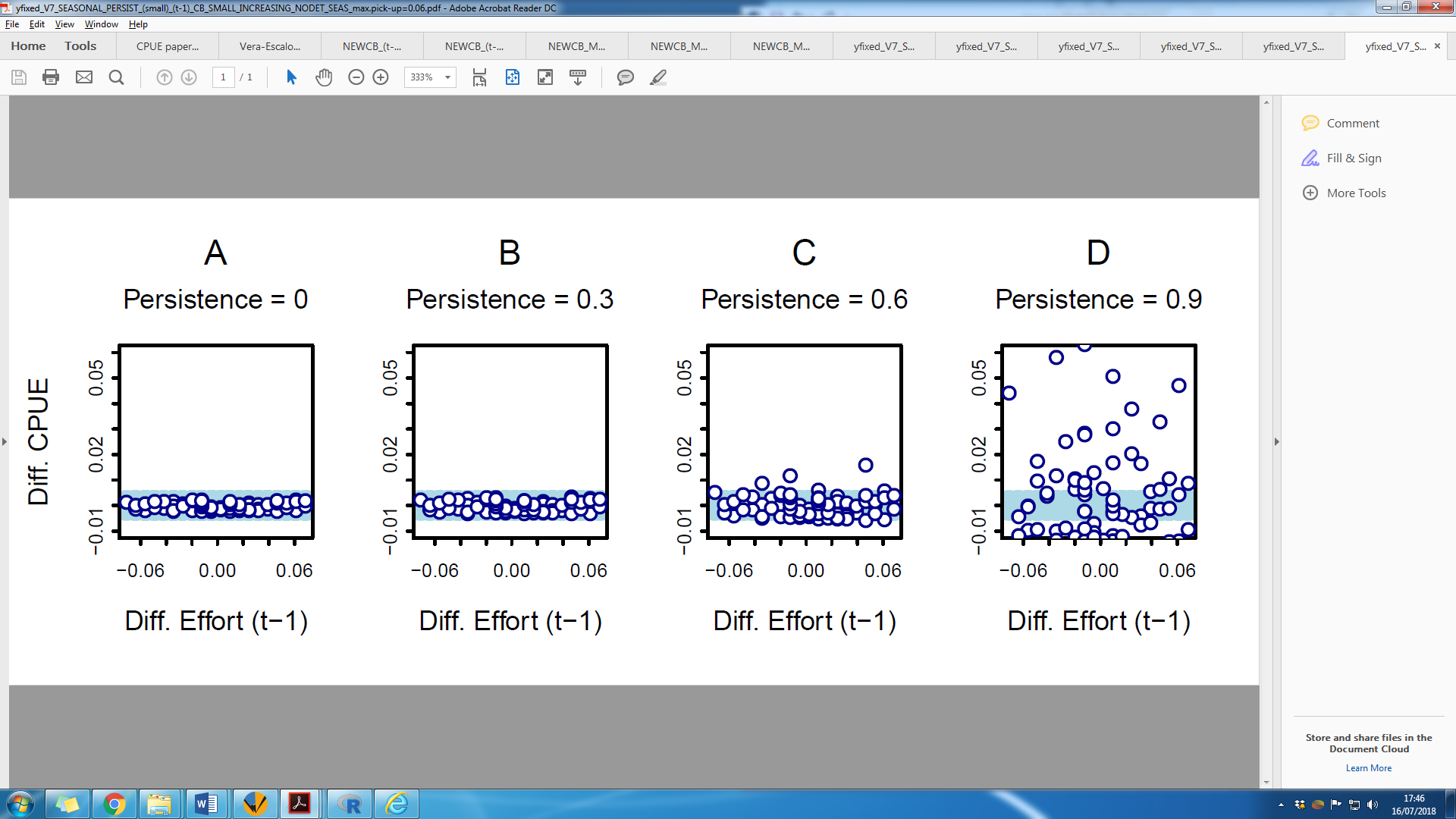


**Fig. S34.** Impact of persistence on differenced CPUE-effort plots (*n*=80). Increasing effort profile, seasonal exogenous change, no deterrence.

**Table S1.** Estimates of *δ* (the total number of snares detected by all patrols per time-step as a fraction of those present in the whole area at *E_max_* (*D_t_*/*A_t_*)) from published studies. *δ* is calculated as (patrol-km/PA size)*detection probability. Conservation patrols in the Russian Far East managed between 250 and 525 patrol-km per month, in protected areas ranging from 834 to 4014km^2^ (Table S1). For two available estimates of detection probability (Hötte et al. 2016; Johnson et al. 2016), *δ* falls between 0.011 and 0.07 (i.e. *δ*≤0.06), meaning that the monthly time-step is ideal for analysis with differenced plots. By contrast, in Nyungwe National Park, Rwanda, patrols were much more frequent (Moore et al. 2018), and using that study’s detection probability of 0.1, *δ* would be approximately 0.17 for data aggregated by month. For that dataset, therefore, we would recommend that patrol data were aggregated in 10-day segments to bring *δ* below 0.06. Note that *δ* should correlate with patrol effort, meaning that the smaller time-steps will only be required when data are numerous, so that the dataset should withstand finer temporal aggregations.

| **Protected Area (PA)** | **Country** | **Maximum patrol-kms per month** | **PA size** | ***δ* (1)*** | ***δ* (2)**** | **Source** |  |
| --- | --- | --- | --- | --- | --- | --- | --- |
| Land of the Leopard National Park | Russia | 300 | 2620 | 0.02 | 0.01 | Hötte et al. (2016) |  |
| Lazovskii State Zapovednik | Russia | 525 | 1240 | 0.07 | 0.04 | Hötte et al. (2016) |  |
| Sikhote-Alin Biosphere Zapovednik | Russia | 450 | 4014 | 0.02 | 0.01 | Hötte et al. (2016) |  |
| Zov Tigra National Park | Russia | 250 | 834 | 0.05 | 0.03 | Hötte et al. (2016) |  |
| Nyungwe National Park | Rwanda | 1666 | 970 | *** | 0.17 | Moore et al. (2018) |  |
| Nam Et-Phou Louey National Protected Area | Laos | 500 | 5950 | 0.01 | 0.01 | Johnson et al. (2016) |  |
| **δ* (1) based on detection rate from (O’Kelly et al. 2018b); each 1km^2^ cell receives 2km of patrol effort, with a detection rate of 0.33 per cell. | | | | | | | |
| ***δ* (2) based on detection rate from (Moore et al. 2018); each 1km^2^ cell receives 1km of patrol effort, with a detection rate of 0.1 per cell. | | | | | | | |
| ***Only δ (2) applied, as it is from this study. It is not fully clear whether the detection probability refers to the probability of detecting each infraction or the probability of detecting *at least one* infraction when there are any number present. If the latter, the estimate of *δ* will be much lower. | | | | | | | |

## Appendix S2. Calculating the ‘correct’ slope in CPUE-effort plots

If deterrence is absent, the correct slope is zero. If deterrence is present, the correct slope is dependent upon various parameters. The number of activities detected is calculated from equations 1 and 2, which are combined as follows:

*A_t_* = *α_t_H* + (*pA_t-1_-D_t-1_*) [1]

*D_t_* = (1-((1-*z*)*^Et^*))*A_t_* [2]

*D_t_* = (1-((1-*z*)*^Et^*))(*α_t_H* + (*pA_t-1_-D_t-1_*))

If deterrence is present, *α_t_* is multiplied by 1-*βE_t-1_*, yielding:

*D_t_* = (1-((1-*z*)*^Et^*))(*α_t_*(1-*βE_t-1_*)*H* + (*pA_t-1_-D_t-1_*))

Without persistence, this simplifies to:

*D_t_* = (1-((1-*z*)*^Et^*))(*α_t_*(1-*βE_t-1_*)*H*)

Since CPUE = *D*/*E*,

CPUE = (1-((1-*z*)*^Et^*))(*α_t_*(1-*βE_t-1_*)*H*)/*E_t_*

To find the y-intercept, set E to zero (this will need to be more like 0.000001 to avoid division by zero) and use default values of *z*, *H*, *α* and *β*:

CPUE*_E_*_=0_ = (1-((1-*z*)*^0^*))(*α_t_*(1-*0*)*H*)/*0*

CPUE*_E_*_=0_ = 0.1053605

Now solve for another value of *E* (assuming constant *E*, such that *E_t_*=*E_t-1_*)^[[1]](#footnote-1)^ to work out the slope. We choose a value approximately mid-way along the effort range (0.05).

CPUE*_E_*_=0.05_ = 0.0966768

The function is effectively linear at these low values of *E*, so we use the form y=mx+c to calculate the slope, yielding:

0.0966768=0.05m+0.1053605

-0.008683539=0.05m

m= -0.1736708

The general formulae are:

y-intercept = ((1-((1-*z*)^0.000001^))*α*(1-*β**0.000001)*H)*/0.000001

Slope = (((((1-((1-*z*)^0.05^))*α*(1-*β**0.05)*H)*/0.05)-( ((1-((1-*z*)^0.000001^))*α*(1-*β**0.000001)*H)*/0.000001))/0.05

The slopes of basic and differenced plots are the same, but the y-intercept of the latter is zero.

1. Note that when the effort profiles are increasing or decreasing, *E* ≠ *E_t-1_*. However, taking into account the difference leads to negligible changes in the calculated ideal slope. [↑](#footnote-ref-1)
